# Oral *Anaerobutyricum soehngenii* augments glycemic control in type 2 diabetes

**DOI:** 10.1016/j.isci.2024.110455

**Published:** 2024-07-05

**Authors:** Ilias Attaye, Julia J. Witjes, Annefleur M. Koopen, Eduard W.J. van der Vossen, Diona Zwirs, Koen Wortelboer, Didier Collard, Elles Marleen Kemper, Maaike Winkelmeijer, Jens J. Holst, Stanley L. Hazen, Folkert Kuipers, Erik S.G. Stroes, Albert K. Groen, Willem M. de Vos, Max Nieuwdorp, Hilde Herrema

**Affiliations:** 1 Department of Vascular Medicine, Amsterdam University Medical Centers, Amsterdam, the Netherlands; 2 Amsterdam Cardiovascular Sciences, Diabetes & Metabolism, Amsterdam, the Netherlands; 3 Amsterdam Gastroenterology Endocrinology Metabolism, Endocrinology, Metabolism and Nutrition, Amsterdam, the Netherlands; 4 Department of Pharmacy and Clinical Pharmacology, Amsterdam University Medical Centers, Amsterdam, the Netherlands; 5 Department of Experimental Vascular Medicine, Amsterdam University Medical Centers, Amsterdam, the Netherlands; 6 NNF Center for Basic Metabolic Research and Department of Biomedical Sciences, Copenhagen University, Copenhagen, Denmark; 7 Department of Cardiovascular and Metabolic Sciences, Lerner Research Institute, Cleveland Clinic, Cleveland, OH 44195, USA; 8 Department of Pediatrics and European Research Institute for the Biology of Ageing (ERIBA), University of Groningen, University Medical Center Groningen, Groningen, the Netherlands; 9 Wageningen University, Wageningen, the Netherlands; 10 Human Microbiome Research Program, Faculty of Medicine, University of Helsinki, Helsinki, Finland

**Keywords:** Health sciences, Human metabolism

## Abstract

This randomized, double-blind, placebo-controlled trial investigated the impact of 14-day *Anaerobutyricum soehngenii* L2-7 supplementation on postprandial glucose levels in 25 White Dutch males with type 2 diabetes (T2D) on stable metformin therapy. The primary endpoint was the effect of *A. soehngenii* versus placebo on glucose excursions and variability as determined by continuous glucose monitoring. Secondary endpoints were changes in ambulatory 24-h blood pressure, incretins, circulating metabolites and excursions of plasma short-chain fatty acids (SCFAs) and bile acids upon a standardized meal. Results showed that *A. soehngenii* supplementation for 14 days significantly improved glycemic variability and mean arterial blood pressure, without notable changes in SCFAs, bile acids, incretin levels, or anthropometric parameters as compared to placebo-treated controls. Although well-tolerated and effective in improving glycemic control in the intervention group, further research in larger and more diverse populations is needed to generalize these findings.

## Highlights

• *Anaerobutyricum soehngenii* supplementation improves glycemic variability in T2D
• *A. soehngenii* supplementation does not affect gut microbiome composition
• Changes in plasma metabolites upon *A. soehngenii* correlate with clinical parameters
• *A. soehngenii* could act as a next-generation therapeutic microbe in T2D

## Graphical abstract

**Figure:**
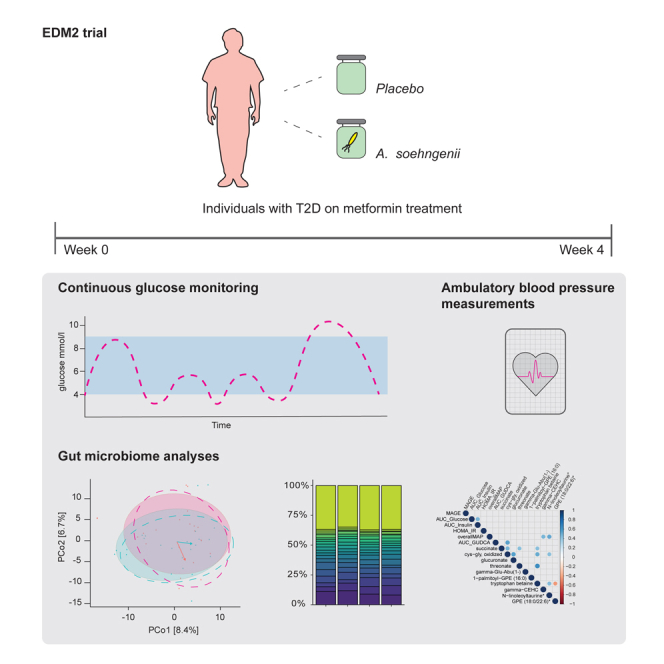

## Introduction

The worldwide prevalence of type 2 diabetes (T2D) is expected to increase to 3% of the adult population by 2050,^1^ highlighting the urgent need for novel insights into this pandemic disease. The pathophysiology of T2D is complex and includes both environmental (such as lifestyle and diet) and genetic factors, with altered gut microbiota composition and functional potential as possible disease modulators.^2^ Over the past two decades, disturbances in the gut microbiota, both in animals and humans, have been linked to the pathophysiology of metabolic diseases such as obesity and T2D.^3^^,^^4^ Obese (insulin-resistant) individuals have been shown to have lower levels of fecal short-chain fatty acid (SCFA)-producing bacteria.^5^ SCFAs are metabolites produced from microbial fermentation of complex fibers, which have been widely implicated in conveying health benefits, such as insulin sensitivity, obesity, and blood pressure regulation, all of which are important elements in the pathophysiology of T2D.^6^^,^^7^^,^^8^ Therapeutic modulation of gut microbiota could serve as an important parameter in both preventive and therapeutic strategies for T2D.

We have previously shown that transplantation of fecal microbiota from lean healthy subjects to insulin-resistant individuals significantly increases insulin sensitivity.^9^ This coincided with an increased relative abundance of bacteria that produced butyrate, an abundant SCFA. Specifically, *Anaerobutyricum soehngenii* (previously known as *Eubacterium hallii)*^10^ increased in the small intestine of treated subjects.^10^ *A. soehngenii* is a well-characterized anaerobe that belongs to the phylum Firmicutes. It can produce butyrate from lactate and acetate in an acidic environment, as found in the small intestine.^11^^,^^12^ In this regard, it is interesting to note that metformin-treated T2D individuals have increased levels of lactate in their feces.^13^ Additionally, lactate levels are positively correlated with blood pressure.^14^ As *A. soehngenii* uses intestinally produced lactate to produce butyrate, which is known to exert beneficial effects on glucose metabolism^11^ and blood pressure,^15^ we hypothesized that adding *A. soehngenii* to metformin treatment in individuals with T2D may improve their cardiometabolic profile.

We previously showed that oral treatment with *A. soehngenii* improves insulin sensitivity and energy expenditure in obese and diabetic *db/db* mice.^16^ In a pilot safety and dose-finding trial in humans, we found that daily oral *A. soehngenii* treatment was safe and well tolerated. Moreover, the relative abundance of the administered *A. soehngenii* was significantly associated with insulin sensitivity, with the highest dose resulting in significant blood pressure reduction.^17^ Furthermore, in a randomized placebo-controlled crossover study in individuals with metabolic syndrome, we showed that a single duodenal infusion of *A. soehngenii* improved 24-h peripheral glycemic control, possibly by modulating intestinal GLP-1 production and/or secondary bile acid levels.^18^

These findings prompted us to study the effect of oral administration of *A. soehngenii* in combination with metformin on (postprandial) glucose excursions, as determined by a wearable flash glucose sensor in individuals with T2D. The secondary endpoints were changes in ambulatory blood pressure, GLP-1 production, circulating metabolites, and (postprandial) excursions of plasma SCFA and bile acids after a standardized meal.

## Results

We included twenty-six White Dutch males with T2D who were on stable metformin monotherapy. Before randomization, one participant was excluded because of acute prednisolone use; during the study, one subject had a malfunctioning FreeStyle Libre (FSL) monitor, and we thus included another subject in order to meet the primary endpoint. A total of 25 individuals completed the trial. Data analysis revealed that one individual had extremely high concentrations of plasma bile acids and an exceptional compositional profile. We excluded this individual from the main data analyses. However, analyses including this subject are presented in Figures S2–S4. Hence, data analyses were performed on 12 placebo-treated and 12 *A. soehngenii*-treated individuals. Baseline body weight, dietary intake, and caloric content did not significantly differ between placebo- and *A. soehngenii-*treated individuals. At baseline, anthropometric and biochemical parameters were similar in both groups (Table S1). Both treatments were well tolerated and no adverse events occurred during the study. The safety laboratory parameters (inflammatory markers and liver parameters) and anthropometric and biochemical parameters were similar in both groups after the intervention (Table 1).

**Table 1:** Four weeks post-intervention characteristics of the EDM2 study

	Placebo (*n* = 12)	Intervention (*n* = 12)	*p* value
BMI (kg/m2)	27.11 (2.53)	27.46 (4.14)	0.81
Weight (kg)	86.53 (12.94)	91.18 (17.26)	0.46
Blood pressure: overall systolic	91.70 (9.24)	99.33 (13.67)	0.15
Blood pressure: overall diastolic	53.00 (7.32)	54.17 (7.84)	0.72
Blood pressure: overall MAP	69.20 (7.41)	70.67 (8.64)	0.68
Fasting glucose (mmol/L)	8.48 (1.99)	8.03 (0.76)	0.46
Insulin (pmol/L)	49.08 (34.26)	42.50 (12.74)	0.54
HbA1c (mmol/mol)	6.71 (0.60)	6.52 (0.57)	0.44
Total Cholesterol (mmol/L)	5.54 (0.69)	5.03 (0.90)	0.14
HDL (mmol/L)	1.30 (0.24)	1.36 (0.32)	0.65
LDL (mmol/L)	3.51 (0.61)	3.02 (0.65)	0.07
Triglycerides (mmol/L)	1.61 (0.69)	1.44 (0.77)	0.58
AST (U/L)	24.42 (5.87)	21.67 (3.08)	0.17
ALT (U/L)	27.42 (11.12)	24.17 (11.58)	0.49
AP (U/L	73.08 (16.73)	69.25 (16.32)	0.58
yGT (U/L)	32.67 (25.40)	26.67 (22.48)	0.55
CRP (mg/mL)	2.01 (1.72)	1.69 (1.42)	0.63
Leukocytes (10^9^/L)	5.97 (1.60)	6.33 (2.12)	0.64
Caloric intake (kcal/day)	1726.68 (418.11)	1749.10 (314.87)	0.89
Fat intake (g/day)	70.23 (30.11)	73.80 (18.62)	0.74
Carbohydrates intake (g/day)	159.15 (58.63)	151.32 (49.60)	0.74
Protein intake (g/day)	87.24 (31.63)	96.05 (26.60)	0.49
Fiber intake (g/day)	18.18 (6.82)	19.72 (5.81)	0.58

Data are expressed as mean ± standard deviation. BMI, body mass index; ALP, alkaline phosphatase; g-GT, gamma glutamyl transferase; ALT, alanine aminotransferase; AST, aspartate aminotransferase; HDLc, high-density lipoprotein cholesterol; LDLc, low density lipoprotein cholesterol; CRP, C-reactive protein.

### *A. soehngenii* supplementation improves glycemic variability

**Figure 1:**
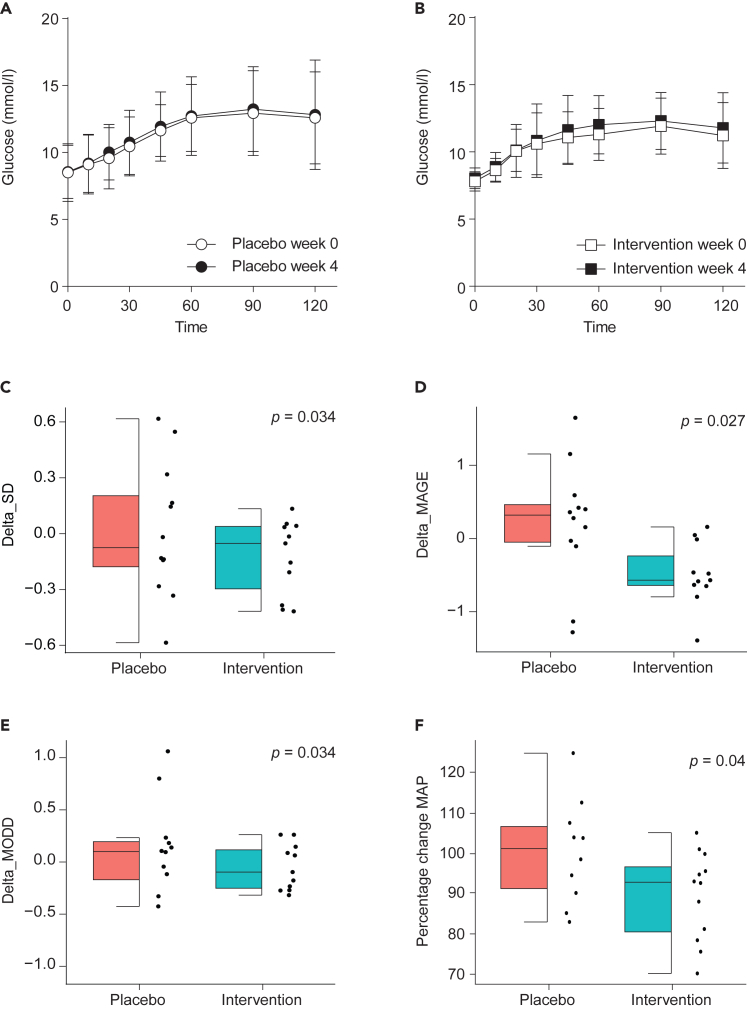
*A. soehngenii* supplementation improves glycemic variability and mean arterial blood pressure Effect of 14 days placebo (A) and *A. soehngenii* (B) supplementation on glucose levels following a 2-h mixed-meal test (ns; data represented as mean ± standard deviation). *A. soehngenii* supplementation significantly improved delta standard deviation (SD) (C), mean amplitude of glycemic excursions (MAGE) represented as median ± interquartile range (D). Mean of daily differences (MODD) (E) and mean amplitude of blood pressure (MAP) (F). Data represented as median ± interquartile range in (C–F), and adjusted for false discovery rate (FDR; Benjamini & Hochberg method). FDR corrected *p* < 0.05 was considered statistically significant. Analyses performed using a linear regression model on delta change, correcting for baseline (delta_change parameter of interest∼Intervention+baseline value parameter of interest).

We previously showed that four weeks of oral supplementation and direct duodenal infusion of *A. soehngenii* in male individuals with metabolic syndrome, who are at increased risk of developing T2D, can affect glycemic control, incretin levels, and blood pressure.^17^^,^^18^ Therefore, we first determined these parameters in metformin-treated T2D individuals orally supplemented with *A. soehngenii* for two weeks. Glucose metabolism was studied using mixed-meal testing (MMT), homeostatic model assessment for insulin resistance (HOMA-IR), and continuous glucose monitoring. Glucose excursions after intake of a standardized mixed meal were similar after two weeks of supplementation with *A. soehngenii* or placebo compared to the baseline (Figures 1A and 1B). Insulin excursions during MMT were unaltered by *A. soehngenii* or placebo supplementation compared to baseline (Figures S5A and S5B). In contrast to our recent findings,^18^ *A. soehngenii* supplementation did not increase GLP1 levels during MMT compared to placebo supplementation (Figures S5C and S5D). In line with observations that glucose and insulin excursions were unaltered, HOMA-IR, as calculated from fasting insulin and glucose levels prior to MMT, remained statistically similar in both groups (Figure S5E).

In addition to an “acute” challenge (MMT) to address glucose tolerance, we equipped subjects with a continuous glucose measuring device (FSL) for 14 days during the run-in phase and then during the 14-day supplementation with *A. soehngenii* or placebo. FSL allows for continuous, long-term, ambulatory glucose measurements, and is increasingly considered a clinically relevant tool to address glucose homeostasis in individuals with T2D or those at risk of developing T2D.^19^ Interestingly, glycemic variability metrics such as standard deviation (SD), mean amplitude of glycemic excursion (MAGE), and mean of daily differences (MODD) were significantly improved in the *A. soehngenii-*treated group compared to the control group. SD was reduced by 7.96% (FDR corrected *p* = 0.034), MAGE was reduced by 16.82% (FDR corrected *p* = 0.027), and MODD was reduced by 3.9% (FDR corrected *p* = 0.034) (Figures 1C–1E). These effects were not driven by extreme outliers as shown by individual changes (Figure S6).

### Mean arterial blood pressure is reduced in individuals supplemented with *A. soehngenii*

SCFA have been associated with beneficial effects on blood pressure; we hypothesized that *A. soehngenii*, potentially via SCFA, would affect blood pressure in our study group. We therefore measured ambulatory blood pressure for 24-h before and after 2 weeks of *A. soehngenii* supplementation and found that mean arterial blood pressure (MAP) was reduced by 10.24% (*p* = 0.04), when looking at percentual change between placebo and intervention group, thereby correcting for potential baseline differences (Figure 1F). This effect is unlikely to be explained by alterations in SCFA levels, since these were unaltered in plasma under both fasting and stimulated (MMT) conditions (Table S2). Fecal lactic acid and butyric acid levels were unaltered in placebo and intervention group (Figure S7). However, it should be noted that SCFA concentrations in the plasma and feces often poorly reflect (local, small intestinal) SCFA production and metabolism.^2^

**Figure 2:**
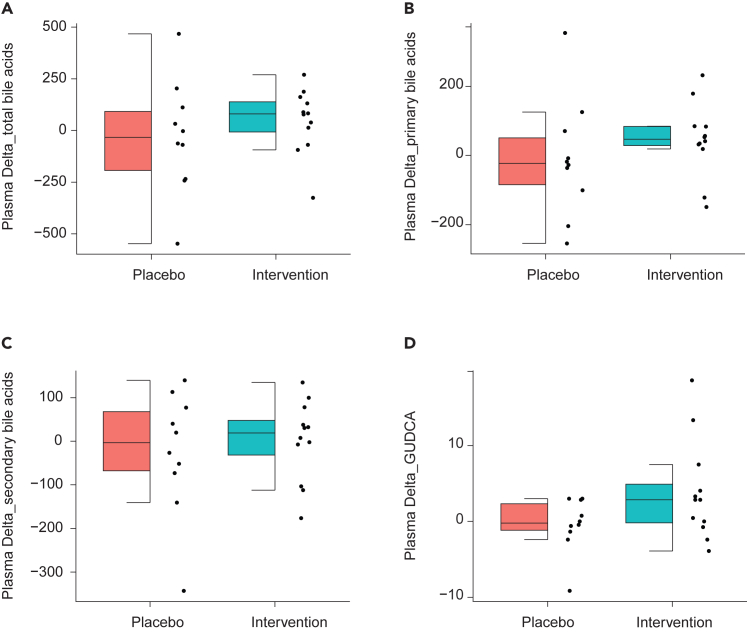
Plasma bile acids remain unaltered after *A. soehngenii* supplementation Effect of 14 days *A. soehngenii* supplementation on delta levels of plasma total bile acid (A), primary bile acids (B), secondary bile acids (C), and glycoursodeoxycholic acid (GUDCA) (D). All data represented as median ± interquartile range, and not significant (ns). A *p* < 0.05 was considered statistically significant. Analyses performed using a linear regression model on delta change, correcting for baseline (delta_change parameter of interest∼Intervention+baseline value parameter of interest).

In our previous safety and dose-response study using *A. soehngenii* in individuals with metabolic syndrome, plasma bile acid levels were found to be altered.^17^ In our current cohort, however, *A. soehngenii* supplementation did not affect (postprandial) plasma bile acid levels (Figures 2A–2D; Table S3). Hence, we were unable to reproduce these findings in individuals with T2D on metformin, since fasting and stimulated (MMT) plasma bile acids were similar in individuals supplemented with *A. soehngenii* and placebo. Fecal bile acids were largely unaltered by *A. soehngenii* supplementation, with the exception of a trend (*p* = 0.08) of increased levels of plasma GUDCA and significantly increased LCA in the intervention group (Table S4).

### *A. soehngenii* abundance and replication activity

**Figure 3:**
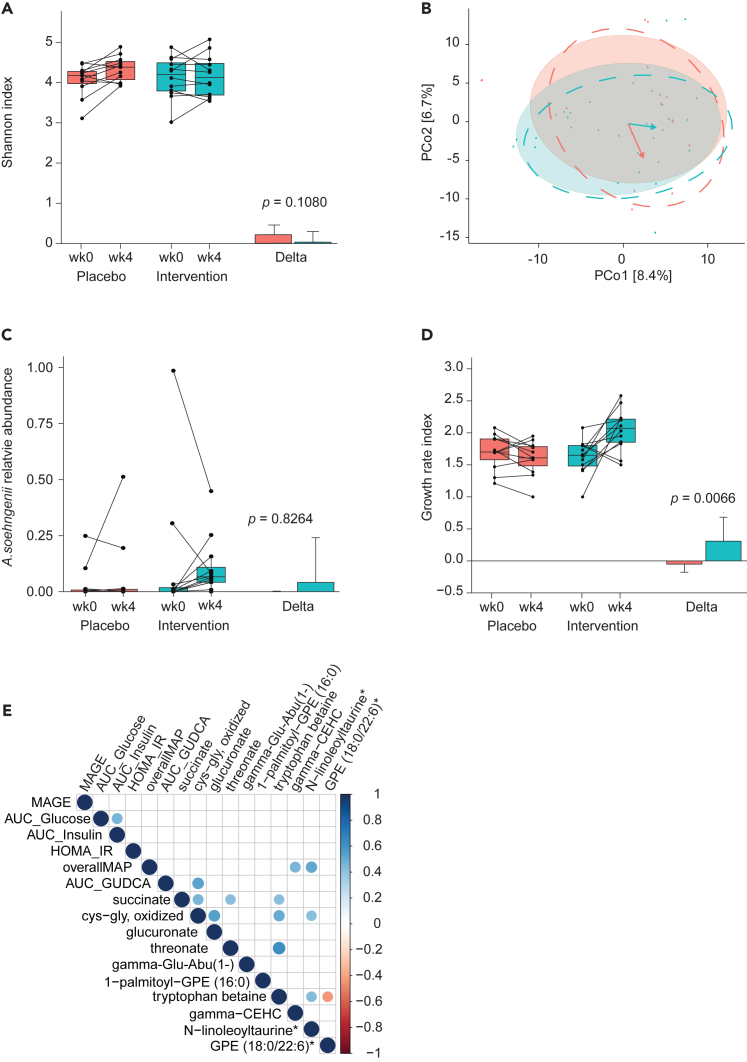
*A. soehngenii* replication activity is increased without altering gut microbiome diversity and composition (A) Effect of *A. soehngenii* supplementation on Shannon index in the two different groups with the delta (mean). A *p* < 0.05 was considered statistically significant. (B) Fecal microbiota composition at baseline and end of the intervention. The PCoA is created via genus-level Aitchison distance. The colors represent the placebo group (red) and the intervention group (green). The symbol represents the pre-intervention samples (circles) and post-intervention samples (triangles). The 80% CI ellipses represent the baseline- (shaded ellipses) and post-intervention groups (dashed ellipses). The colored arrows represent the intervention effects with its length and direction corresponding to the shift in group centroid coordinates from baseline to endpoint for each treatment arm (re-scaled ×4 and re-centered at baseline global centroid). (C) Relative abundances of *A. soehngenii* following placebo or *A. soehngenii* supplementation. (D) Replication activity of the *A. soehngenii* between the placebo and intervention group over time with the delta values (mean). (E) Delta spearman correlations within and between metabolites and clinical parameters. A blue circle indicates a significant positive correlation, whereas a red circle indicates a significant inverse correlation. *p* values are not adjusted for multiple test companions.

We performed shotgun metagenomics to determine whether *A. soehngenii* supplementation altered the gut microbiome composition and diversity. Alpha diversity (Shannon index) (Figure 3A) and beta diversity (Aitchison) (Figure 3B) were similar between *A. soehngenii*- and placebo-treated groups. Taxonomic analyses did not reveal significant changes in bacterial groups at phylum and genus levels (Figure S8). The relative abundance of *A. soehngenii* showed an increasing trend in the intervention group but was not significant (Figure 3C). This could be attributed to potential competition for the niche in the gastrointestinal (GI) tract between our supplemented *A. soehngenii* L2-7 and the endogenous *A. soehngenii* that was already present in two subjects receiving the intervention during the allocation phase (Figure S9). Indeed, our intervention strain L2-7 completely replaced the native strain in one subject, resulting in a relative abundance comparable to that of other subjects receiving the intervention (Figure 3C). For the other subject, competition seemed evident, leading to a severe decrease in the relative abundance of the native strain (Figure S9). To circumvent the confusion of the relative abundance due to this potential competition for the niche in the GI tract, we assessed the growth rate index of the total *A. soehngenii*. This was significantly increased in individuals supplemented with *A. soehngenii,* implying the presence of viable *A. soehngenii* in the gut upon treatment (*p* = 0.0066, Figure 3D).

We then addressed whether the plasma metabolome of individuals that received two weeks of supplementation with *A. soehngenii* differed from the plasma metabolome of placebo-supplemented controls. We identified the top 10 plasma metabolites that were altered by the intervention, and performed correlation analyses with clinical markers (Figure 3E). This study identified positive correlations between gamma-CEHC and N-linoleoyltaurine levels and MAP. No negative correlations were identified between the plasma metabolites and clinical markers.

## Discussion

In this randomized, double-blind, placebo-controlled trial, we showed that 14 days of oral treatment with *A. soehngenii* L2-7 alters glucometabolic parameters in T2D subjects receiving stable metformin therapy. *A. soehngenii* supplementation significantly improved glycemic variability based on real-time measurements with continuous glucose monitoring. Moreover, we identified the changes in plasma metabolites and their correlation with several metabolic parameters.

Previous rodent and human studies have shown that *A. soehngenii* can positively influence glucose metabolism.^16^^,^^17^^,^^18^ A study in obese mice showed that daily supplementation of *A. soehngenii,* through oral gavage for four weeks, improved insulin sensitivity and affected bile acid metabolism.^16^ The positive effect of *A. soehngenii* supplementation on glucose metabolism was also confirmed in two human intervention studies, both focusing on treatment-naive subjects with metabolic syndrome.^17^^,^^18^ In a recent study, direct intestinal infusion of a single dose of *A. soehngenii* in the duodenum led to an improvement in glycemic variability, as measured via continuous glucose monitoring.^18^ We speculate that this improvement may be modulated through changes in SCFA-GLP-1 levels and/or secondary bile acids in individuals with metabolic syndrome. Here, we show that *A. soehngenii* supplementation lowers glycemic variability by lowering SD, MAGE, and MODD. These parameters are well-established glucose variability metrics that correlate strongly with each other, poor metabolic health, and more cardiovascular events.^20^^,^^21^^,^^22^^,^^23^ Despite being able to replicate the beneficial effects of *A. soehngenii* on glucose variability in our current study population, we were unable to find clinically relevant changes in GLP-1, SCFAs, or secondary bile acids.

It is important to note that previous human studies have focused on treatment-naive metabolic syndrome subjects, whereas this study investigated the additional glucose-lowering effect of *A. soehngenii* supplementation in T2D subjects on metformin therapy, a well-known modulator of the gut microbiota.^24^ GLP1 excursions were 2-fold higher in T2D individuals on metformin than in individuals with metabolic syndrome. This is potentially due to metformin-mediated effects on GLP1 secretion^25^ and might explain why we did not observe GLP1 effects of *A. soehngenii* in addition to metformin effects on GLP1, next to a more indirect way of treatment by ingestion instead of direct duodenal infusion.

We hypothesized that beneficial actions of *A. soehngenii* could be mediated by butyric acid acid production derived from increased lactic acid levels in T2D individuals on metformin. Lactate utilization of this bacterium was in large determined in *in vitro* settings and our study served to test if we could indeed find changes in lactate-butyrate in humans with T2D on metformin and co-supplemented with *A. soehngenii*. We were unable to detect changes in fasting or postprandial SCFA levels in plasma. Fecal lactic acid and butyric acid levels were also unaltered in feces in both the placebo and intervention group. It is notoriously difficult to interpret plasma and fecal SCFA levels in humans, considering the rapid local turnover of SCFA in the gut.

Previous studies have shown that *A. soehngenii* supplementation can not only increase total plasma secondary bile acid levels after oral supplementation, but also following direct duodenal infusion.^17^^,^^18^ These observations are important because bile acid metabolism, gut microbiota composition, and cardiometabolic health are closely associated.^26^ However, the current study does not support previous findings of a significant increase in plasma secondary bile acid levels in individuals treated with *A. soehngenii*. However, we observed a trend (*p* = 0.08) for an increased level of plasma GUDCA, a secondary bile acid that has been found to exert protective effects against diet-induced insulin resistance in preclinical models.^27^ Of note, metformin treatment has been shown to affect the gut microbiota and increase the levels of GUDCA, which might abrogate further increases in GUDCA in the current study population.^28^

*A. soehngenii* supplementation did not induce large-scale changes in alpha or beta diversity in this study, which is in accordance with previous studies.^18^ Using advanced replication analysis, we confirmed that *A. soehngenii* engraftment was successful. Nevertheless, engraftment was not similar among all the participants in the treatment group. Two individuals in the treatment group had a similar *A. soehngenii* strain present at baseline, which might have inhibited the L2-7 strain from engrafting in the intestine. This finding is in accordance with a recent study showing that the engraftment of exogenous strains is likely unsuccessful when (closely) related strains are already present in the gut.^29^ However, in two other individuals, engraftment was inferior to that in other participants without prior presence of *A. soehngenii* strains at baseline. Although we monitored compliance and all participants returned the complete number of empty vials, we cannot exclude poor compliance in these individuals.

Gut microbiota-derived metabolites are thought to be key players and potential modifiable factors in the pathophysiology of T2D.^2^ Therefore, this study investigated the effects of *A. soehngenii* supplementation on (gut-derived) plasma metabolites. Using untargeted semi-quantitative metabolomics, we identified several plasma metabolite changes that were altered by *A. soehngenii* treatment combined with metformin, which were subsequently correlated with clinical markers. Positive correlations were identified between plasma gamma-CEHC, N-linoleoyl taurine levels, and MAP. No negative correlations were identified between the plasma metabolites and clinical markers. The metabolites that correlate with MAP have been scarcely described in the literature and are linked to fatty acid metabolism (N-linoleoyl taurine^30^) and vitamin E metabolism (gamma-CEHC^31^). As such, the association of N-linoleoyl taurine and reduced blood pressure is a new finding of this study and it is tempting to speculate that this compound has a similar action as taurine that is known to reduce hypertension in human and animal models due to its structural similarity.^32^ Similarly, gamma-CEHC has been shown to have anti-inflammatory properties rationalizing the observed correlation of this compound and blood pressure.^33^ However, it is important to note that the identified metabolites are not well studied, and correlations found need to be interpreted with caution. Further research is needed on these plasma metabolites, with a specific emphasis on the clinical parameters with which we found correlations.

In conclusion, this is the first randomized, double-blind, placebo-controlled clinical trial to study the augmenting effects of two weeks of single bacterial strain *A. soehngenii* supplementation in T2D subjects on stable metformin monotherapy. We found that *A. soehngenii* supplementation was well-tolerated and improved glucose variability and MAP. The results of this study indicate that *A. soehngenii* can act as a next-generation therapeutic microbe that can improve metabolic health following metformin therapy. Larger clinical trials are therefore warranted to investigate this augmenting effect in a more sex and ethnically diverse population and to determine if *A. soehngenii* supplementation for a longer duration has durable beneficial metabolic effects.

### Limitations of the study

Our study had several limitations. First, to show proof of concept effects, T2D participants were treated for a relatively short period of time. Long-term studies in larger groups of participants will have to show if the observed effect of *A. soehngenii* on glucometabolic control is long-lasting. Second, this pilot study aimed to provide proof-of-concept insight into the potential synergy between next-generation probiotics and oral pharmacological treatment; thus, the size of our study was relatively small. Third, all participants received oral metformin therapy, which could have had a confounding effect as metformin also altered the gut microbiota composition.^34^ However, metformin is among the first-choice therapies for T2D; therefore, the selection of individuals included in this trial makes the results more generalizable to the patient population itself.^35^ The fact that this study already provided significant findings following short-term *A. soehngenii* supplementation on glucometabolic control indicates the potential of *A. soehngenii* as an add-on therapy in the standard of care for T2D.

## STAR★Methods

### Key resources table

**Table:** 

REAGENT or RESOURCE	SOURCE	IDENTIFIER
**Bacterial and virus strains**		

*A. soehngenii* L2-7	N/A	N/A

**Biological samples**		

Human plasma metabolomics data	This study (PI Dr. H.Herrema)	N/A
Human fecal metagenomics data	This study (PI Dr. H.Herrema)	N/A
Human plasma and fecal bile acid data	This study (PI Dr. H.Herrema)	N/A
Human plasma and fecal SCFA data	This study (PI Dr. H.Herrema)	N/A

**Chemicals, peptides, and recombinant proteins**		

		Klik of tik om tekst in te voeren.

**Critical commercial assays**		

DNA extraction kit	QIAamp DNA Mini kit	N/A

**Software and algorithms**		

open software program R version 4.2.1	( R Core Team (2022). R: A Language and Environment for Statistical Computing. R Foundation for Statistical Computing, Vienna, Austria. URL https://www.R-Project.org/., n.d.)	https://www.R-project.org/
MEDUSA pipeline	N/A	(Karlsson et al. 2014)

**Other**		

Freestyle Libre Glucose Monitoring System	ABBOTT DIABETES CARE IN FRANCE Abbott France S.A.S. Abbott Diabetes Care 94528 Rungis Cedex France	version 1.0
Nutridrink Compact Protein	Nutricia Advanced Medical Nutrition, Amsterdam, the Netherlands	Strawberry compact protein
Food diary log “Mijn Eetmeter”	Stichting Voedingscentrum Nederland	N/A

### Resource availability

#### Lead contact

Further information should be directed to and will be fulfilled by the lead contact Dr. H.J. Herrema (h.j.herrema@amsterdamumc.nl).

#### Materials availability

This study did not generate new unique reagents or materials.

#### Data and code availability

Metagenome and metabolome data have been reposited in the European Genome-phenome Archive (EGA) with accession number EGAS50000000415. https://ega-archive.org/studies/EGAS50000000415. Data reported in this paper will be shared by the lead contact upon reasonable request.

This paper does not report original code.

Any additional information required to reanalyze the data reported in this paper is available from the lead contact upon reasonable request.

### Experimental model and study participant details

#### Study population and ethics approval

Twenty-six white Dutch males with T2D on metformin monotherapy were recruited through local newspaper advertisements. To be eligible to participate, all individuals had to be on a stable dose of metformin (*i.e.*, no changes in the last three months), two or three times daily 500 mg or twice daily 850 mg, with no other medication use. The exclusion criteria included smoking, moderate to heavy alcohol use (defined as >12 g/day), a history of cardiovascular events, cholecystectomy, overt untreated gastrointestinal disease or abnormal bowel habits, elevated liver enzymes >2.5 times the upper limit of the normal range, prolonged compromised immunity, and the use of antibiotics in the past three months. Only male individuals were included, because changes in female hormone concentrations in (postmenopausal) women have a disturbing effect on insulin sensitivity.^36^ Participants were requested to maintain their usual physical exercise and dietary patterns during the study and to keep an online nutritional diary to monitor food intake (https://mijn.voedingscentrum.nl/nl/eetmeter/).

The study protocol was reviewed and approved by the local Institutional Review Board of the Amsterdam University Medical Center (Amsterdam UMC, Amsterdam, Netherlands) and conducted in accordance with the Declaration of Helsinki and CONSORT guidelines. The study was registered in July 2018 at the Dutch Clinical Trial Register (registration number NL7121), which is a primary registry included in the International Clinical Trial Registry Platform (ICTRP). All the participants provided written informed consent. The study protocol can be found as Data S1.

### Method details

#### Study design

This was a randomized double-blind placebo-controlled single-center study. Figure S1 shows an overview of the study design. During the run-in phase, the participants continued their stable dosage of metformin, and their glucose levels were continuously measured using a disposable flash glucose sensor (FreeStyle Libre, FSL, Abbott, USA), which has been validated in previous studies.^37^^,^^38^ Afterwards, participants were randomized to receive either 14 days of once-daily oral treatment with *A. soehngenii* L2-7 or placebo. Participants in the treatment arm ingested daily one 10 ml vial of *A. soehngenii* in phosphate-buffered saline (PBS) with 10% glycerol, whereas participants in the placebo arm received 10 ml of PBS with 10% glycerol. We recently described the anaerobic production of *A. soehngenii*^39^ in detail. The earlier Most Probable Number (MPN) analysis for quantifying live and anaerobic cells has been complemented with high-throughput determination based on flow cytometry according to ISO 19344 (ISO 2015).^40^ Based on the latter method, the daily dose was estimated to be 10^10^ active cells. The viability of *A. soehngenii L2-7* in randomly selected tubes (stored at -80°C at the AMC Department of Clinical Pharmacy, conforming to good clinical practice (GCP)) was regularly tested, while purity was assessed by testing the growth/presence of various pathogens/contaminants.^39^ Consort checklist is presented in Data S2.

Participants underwent a two-hour mixed meal test, conducted as previously described,^41^ before and after the intervention, with baseline blood sampling from an intravenous catheter in a distal arm vein. Thereafter, participants immediately ingested a liquid meal solution (Nutridrink; Nutricia Advanced Medical Nutrition, Amsterdam, Netherlands) containing 600 kcal (35% fat, 49% carbohydrates, and 16% proteins), and for the next two hours blood samples were drawn to determine postprandial glucose, insulin, and GLP-1 excursions. Afterwards, the participants were equipped with a disposable flash glucose sensor (FreeStyle Libre, FSL, Abbott, USA), which was worn for 14 days before and 14 days during the intervention to continuously monitor (postprandial) glucose excursions. In addition, participants received a blood pressure monitor to measure ambulatory blood pressure for 24-hours before and after treatment. Finally, feces were collected from all participants at baseline, week two and week four of the study. Compliance was determined by having participants return empty vials and keep track of daily intake of either the placebo or *A. soehngenii L2-7* via diaries.

#### Randomization and blinding

The study participants and all trial physicians were blinded to the treatment until the completion of the trial. The participants were randomized using a randomization list produced by the clinical pharmacy of the Amsterdam UMC.

### Quantification and statistical analysis

#### Power calculation and statistical analyses

Based on previous studies^9^^,^^18^ and the hypothesized peak difference in postprandial glucose excursion, we calculated that we needed 12 subjects per arm to detect a significant difference in this trial. This number was based on a significance level of 0.05 and 80% power, and was calculated using an online power calculation (www.biomath.info/power). Delta changes before and after the intervention were calculated and appropriate (non) parametric tests were performed. All statistical analyses were performed using the open software program, R (version 4.2.1). FreeStyle Libre data were processed using the CGDA package.^42^ Statistical significance was set at P < 0.05. Primary endpoint was changes in glycaemic variability, as determined via measurements of standard deviation, mean amplitude glycaemic excursions (MAGE) and mean of daily differences (MODD). Secondary endpoints were effects of *A.Soehngenii* on blood pressure regulation, glucose and insulin levels following an OGTT, bile acid profiles, metabolomic profiles and gut microbiota composition.

#### Biochemical, microbiome and metabolome analyses

##### Plasma SCFA measurement

Plasma SCFAs were measured at Cleveland Clinic (OH, USA). Collected plasma heparin samples were directly frozen at -80°C and were used for gas chromatography coupled to tandem mass spectrometry (GC-MS/MS). For a brief, step-by-step description see Koopen et al.^18^

##### Plasma incretin measurement

Two-hour mixed meal tests were performed in all individuals and plasma incretin levels were determined in the collected postprandial samples as described previously.^9^ Concentrations of total plasma GLP-1 were measured with the use of ELISA kits (cat no. 10-1258-01 and 10-1278-01, Mercodia, Sweden). The quality controls provided by the manufacturer were within the allowed limits and all samples of one individual were measured in the same assay run to reduce variability.

##### Plasma and fecal bile acid measurement

Ultra high-performance liquid chromatography-tandem mass spectrometry (UPLC-MS/MS) was used to measure the plasma concentrations of the primary, secondary, conjugated and unconjugated bile acids ursodeoxycholic acid (UDCA), cholic acid (CA), glycoursodeoxycholic acid (GUDCA), glycocholic acid (GCA), tauroursodeoxycholic acid (TUDCA), taurocholic acid (TCA), chenodeoxycholic acid (CDCA), deoxycholic acid (DCA), glycochenodeoxycholic acid (GCDCA), glycodeoxycholic acid (GDCA), taurochenodeoxycholic acid (TCDCA), taurodeoxycholic acid (TDCA), lithocholic acid (LCA), taurolithocholic acid (TLCA), glycolithocholic acid (GLCA) as described previously.^43^ For a brief, step- by step description please see elsewhere^44^^,^^45^ (4,5). Fecal samples (from 24hr fecal collection) were thawed and homogenized with distilled water (1:1; w/w). A total of 10 mL of fecal homogenate was dispensed into a 10 mL plastic tube. Bile salts were extracted and quantified as their methyl-trimethylsilyl derivatives.^46^ Bile salts were analyzed by gas chromatography (Agilent 6890) using a CPSil 19 capillary column (25 m x 0.25 mm x 0.2 um; Chromepack).

##### Plasma metabolite profiling

Global untargeted targeted metabolite profiling was performed at both timepoints on the sampled blood by Metabolon (Morrisville, NC, USA) using ultra high-performance liquid chromatography coupled to tandem mass spectrometry, as previously described.^47^ Metabolites that were either all zero or constant, were omitted. Next, the values of each metabolite across all samples were rescaled to 1, in which missing values were imputed with the lowest present value for its respective metabolite.

##### Fecal sample collection and analyses

Both fresh morning stool samples and 24-hour stool samples were directly frozen at -20°C after collection. Stool samples of all participants were taken at 0, 2 and 4 weeks after initiation of the study and transferred in frozen form to the study center, where the samples were stored at -80°C until use.

##### Fecal SCFA measurement

Short chain fatty acid (butyrate, acetate, propionate) levels were measured in fecal samples at Cleveland Clinic. Fresh morning stool samples were directly frozen at -20°C after collection and gas chromatography coupled to tandem mass spectrometry detection (GC-MS/MS) was used for the measurement of SCFA levels in these samples. To start, 20-100 mg of the sample was mixed with internal standards and freeze dried. Then HCl was added to the samples with the use of diethyl ether extraction SCFAs were extracted. The derivatization agent N-tert-butyldimethylsilyl-N- methyltrifluoroacetamide (Sigma-Aldrich, Stockholm, Sweden) was added to the collected organic supernatant and samples were incubated at room temperature overnight. Using a gas chromatograph (Agilent Technologies 7890A, Santa Clara, California, USA) coupled to a mass spectrometer (Agilent Technologies 5975C) SCFAs were quantified. SCFA standards were attained from Sigma-Aldrich (Stockholm, Sweden).

##### Gut microbiota profiling

Fecal samples were isolated as previously described^48^ and analysed for microbiota composition using shotgun metagenomic sequencing (Novogene, Cambridge, UK). Raw reads were checked and quality- filtered using fastp (v.0.20.0). Here, the adapter was detected and removed, 5 bp in front for read1 was trimmed, and sliding window quality trimming was applied (with a window width of 4 bp and threshold Q-score of 15). After trimming and adapter removal, reads shorter than 70 bp were removed. Paired-end reads that passed the quality filtering were mapped against the human genome (hg19) using Bowtie 2 (v.2.4.1). SAMtools (v.1.9), sambamba (v.0.7.1) and BEDtools (v.2.27.1) were used to remove reads that were mapped to the human genome. Unmapped reads were subsampled to 20 milion reads. The remaining high-quality, non-human reads were fed to the MetaPhlAn pipeline (v.4.0.2) for taxonomic profiling^49^ (9).

##### Growth rates of A. soehngenii from metagenomic sequencing data

The growth of the A. soehngenii was determined using the growth rate index algorithm as previously described.^50^ In short, the principle is based on that most bacteria harbour a circular chromosome, in which bi-directional replication takes place from the origin of replication to the terminus. Hereby, in a fast-growing bacterium, the copy number of DNA will be larger at the origin of replication when compared to the terminus.

Differentiating the administered A. soehngenii strain from endogenous Anaerobutyricum spp.

The intervention strain A. soehngenii L2-7 was distinguished from endogenous A. soehngenii via single nucleotide variants (SNVs). For each subject, a unique subset of SNVs was used to discriminate between the administered A. soehngenii L2-7 and endogenous strains. Reads were first processed as described in “Gut microbiota profiling”. After extracting A. soehngenii reads using the reference genome of the A. soehngenii L2-7, GATKs HaplotypeCaller was applied, with a ploidy assumption of 10. The pipeline consists of multiple filters. The first filters include a variant confidence standardized by depth (QD < 2.00), Fisher exact test for strand bias (FS > 60), strand odds ratio test for strand bias (SOR > 3.00), Mapping quality (MQ < 32.00), Rank sum test for mapping qualities of reference versus alternative reads (MQRankSum < -12.5) and rank sum test for relative positioning of reference versus alternative alleles within reads (ReadPosRankSum < -8.00). The second filter hereafter requires the SNV to have a depth of at least 6 reads per sample. The third filter is based on the removal of SNVs found in samples not containing A. soehngenii (e.g., spurious mapping locations such as mobile elements, 16s region).

##### Machine learning analyses

We applied an Extremely Randomized Trees machine learning classification algorithm to identify which parameters (relative changes at week 2 (allocation) and week 4 (end of study)) best distinguished between the intervention- and placebo group. Prior to applying the model, metabolites were filtered to reduce dimensionality. First, metabolites that were zero or constant for all subjects, were omitted. Hereafter, the Rosner’s test for outliers was applied, omitting features that have one outlier. Features with the largest variance were included. Lastly, a univariate feature selection (SelectPercentile) was applied to include 50 metabolites in the model.

Within the machine learning simulation, all models were constructed with the same stability selection procedure to ensure robustness of the results and prevent overfitting. In total, the dataset is split up 20 times. Within these 20 random subsets, 85% of the subjects were included. Next, LeaveOneOut cross-validation was applied where the training set included all samples except for one, in which this one sample left out was included in the test set. Within the training set, the hyperparameters of extremely randomized trees model were found by performing a randomised search with a three-fold cross-validation, based on 90% of the training set and validated on the remaining 10%. The parameter grid on which the randomised search was applied with the number of parameter settings tried was 10. Performance of the different models was estimated via an area under the curve (AUC) of the test dataset to distinguish the intervention group from the placebo group. The importance of each gut microbiota in the models was extracted and was based on the permutation importance. This machine learning pipeline was implemented in python (v3.7.7), using the scikit-learn (v0.23.1) package.

### Additional resources

The study was registered at the Dutch Clinical Trial Register (registration number NL7121), which is a primary registry included in the International Clinical Trial Registry Platform (ICTRP).

## References

[1] Boyle J.P., Thompson T.J., Gregg E.W., Barker L.E., Williamson D.F. (2010). Projection of the year 2050 burden of diabetes in the US adult population: dynamic modeling of incidence, mortality, and prediabetes prevalence. Popul. Health Metr..

[2] Herrema H., Niess J.H. (2020).

[3] Ridaura V.K., Faith J.J., Rey F.E., Cheng J., Duncan A.E., Kau A.L., Griffin N.W., Lombard V., Henrissat B., Bain J.R. (2013). Gut Microbiota from Twins Discordant for Obesity Modulate Metabolism in Mice. Science.

[4] Pedersen H.K., Gudmundsdottir V., Nielsen H.B., Hyotylainen T., Nielsen T., Jensen B.A.H., Forslund K., Hildebrand F., Prifti E., Falony G. (2016). Human gut microbes impact host serum metabolome and insulin sensitivity. Nature.

[5] Karlsson F.H., Tremaroli V., Nookaew I., Bergström G., Behre C.J., Fagerberg B., Nielsen J., Bäckhed F. (2013). Gut metagenome in European women with normal, impaired and diabetic glucose control. Nature.

[6] Vallianou N.G., Geladari E., Kounatidis D. (2020). Microbiome and hypertension: where are we now?. J. Cardiovasc. Med..

[7] Verhaar B.J.H., Collard D., Prodan A., Levels J.H.M., Zwinderman A.H., Backhed F., Vogt L., Peters M.J.L., Muller M., Nieuwdorp M. (2020).

[8] Flint H.J., Duncan S.H., Scott K.P., Louis P. (2015). Links between diet, gut microbiota composition and gut metabolism. Proc. Nutr. Soc..

[9] Kootte R.S., Levin E., Salojärvi J., Smits L.P., Hartstra A.V., Udayappan S.D., Hermes G., Bouter K.E., Koopen A.M., Holst J.J. (2017). Improvement of Insulin Sensitivity after Lean Donor Feces in Metabolic Syndrome Is Driven by Baseline Intestinal Microbiota Composition. Cell Metab..

[10] Shetty S.A., Zuffa S., Bui T.P.N., Aalvink S., Smidt H., De Vos W.M. (2018). Reclassification of eubacterium hallii as Anaerobutyricum hallii gen. nov., comb. nov., and description of Anaerobutyricum soehngenii sp. nov., a butyrate and propionate-producing bacterium from infant faeces. Int. J. Syst. Evol. Microbiol..

[11] Duncan S.H., Louis P., Flint H.J. (2004). Lactate-Utilizing Bacteria, Isolated from Human Feces, That Produce Butyrate as a Major Fermentation Product. Appl. Environ. Microbiol..

[12] Schwab C., Ruscheweyh H.-J., Bunesova V., Pham V.T., Beerenwinkel N., Lacroix C. (2017). Trophic Interactions of Infant Bifidobacteria and Eubacterium hallii during L-Fucose and Fucosyllactose Degradation. Front. Microbiol..

[13] Wu H., Esteve E., Tremaroli V., Khan M.T., Caesar R., Mannerås-Holm L., Ståhlman M., Olsson L.M., Serino M., Planas-Fèlix M. (2017). Metformin alters the gut microbiome of individuals with treatment-naive type 2 diabetes, contributing to the therapeutic effects of the drug. Nat. Med..

[14] Crawford S.O., Ambrose M.S., Hoogeveen R.C., Brancati F.L., Ballantyne C.M., Young J.H. (2008). Association of lactate with blood pressure before and after rapid weight loss. Am. J. Hypertens..

[15] Cookson T.A. (2021). Bacterial-Induced Blood Pressure Reduction: Mechanisms for the Treatment of Hypertension via the Gut. Front. Cardiovasc. Med..

[16] Udayappan S., Manneras-Holm L., Chaplin-Scott A., Belzer C., Herrema H., Dallinga-Thie G.M., Duncan S.H., Stroes E.S.G., Groen A.K., Flint H.J. (2016). Oral treatment with Eubacterium hallii improves insulin sensitivity in db/db mice. NPJ Biofilms Microbiomes.

[17] Gilijamse P.W., Hartstra A.V., Levin E., Wortelboer K., Serlie M.J., Ackermans M.T., Herrema H., Nederveen A.J., Imangaliyev S., Aalvink S. (2020). Treatment with Anaerobutyricum soehngenii: a pilot study of safety and dose–response effects on glucose metabolism in human subjects with metabolic syndrome. NPJ Biofilms Microbiomes.

[18] Koopen A., Witjes J., Wortelboer K., Majait S., Prodan A., Levin E., Herrema H., Winkelmeijer M., Aalvink S., Bergman J.J.G.H.M. (2022). Duodenal Anaerobutyricum soehngenii infusion stimulates GLP-1 production, ameliorates glycaemic control and beneficially shapes the duodenal transcriptome in metabolic syndrome subjects: a randomised double-blind placebo-controlled cross-over study. Gut.

[19] Danne T., Nimri R., Battelino T., Bergenstal R.M., Close K.L., DeVries J.H., Garg S., Heinemann L., Hirsch I., Amiel S.A. (2017). International consensus on use of continuous glucose monitoring. Diabetes Care.

[20] Akasaka T., Sueta D., Tabata N., Takashio S., Yamamoto E., Izumiya Y., Tsujita K., Kojima S., Kaikita K., Matsui K., Hokimoto S. (2017). Effects of the Mean Amplitude of Glycemic Excursions and Vascular Endothelial Dysfunction on Cardiovascular Events in Nondiabetic Patients With Coronary Artery Disease. J. Am. Heart Assoc..

[21] Zhou Z., Sun B., Huang S., Zhu C., Bian M. (2020).

[22] Suh S., Kim J.H. (2015). Glycemic Variability: How Do We Measure It and Why Is It Important?. Diabetes Metab. J..

[23] Tang X., Li S., Wang Y., Wang M., Yin Q., Mu P., Lin S., Qian X., Ye X., Chen Y. (2016). Glycemic variability evaluated by continuous glucose monitoring system is associated with the 10-y cardiovascular risk of diabetic patients with well-controlled HbA1c. Clin. Chim. Acta.

[24] Lee H., Lee Y., Kim J., An J., Lee S., Kong H., Song Y., Lee C.-K., Kim K. (2018). Modulation of the gut microbiota by metformin improves metabolic profiles in aged obese mice. Gut Microb..

[25] Bahne E., Sun E.W.L., Young R.L., Hansen M., Sonne D.P., Hansen J.S., Rohde U., Liou A.P., Jackson M.L., de Fontgalland D. (2018). Metformin-induced glucagon-like peptide-1 secretion contributes to the actions of metformin in type 2 diabetes. JCI Insight.

[26] Ridlon J.M., Kang D.J., Hylemon P.B., Bajaj J.S. (2014).

[27] Cheng L., Chen T., Guo M., Liu P., Qiao X., Wei Y., She J., Li B., Xi W., Zhou J. (2021). Glycoursodeoxycholic acid ameliorates diet-induced metabolic disorders with inhibiting endoplasmic reticulum stress. Clin. Sci..

[28] Sun L., Xie C., Wang G., Wu Y., Wu Q., Wang X., Liu J., Deng Y., Xia J., Chen B. (2018). Gut microbiota and intestinal FXR mediate the clinical benefits of metformin. Nat. Med..

[29] Schmidt T.S.B., Li S.S., Maistrenko O.M., Akanni W., Coelho L.P., Dolai S., Fullam A., Glazek A.M., Hercog R., Herrema H. (2022). Drivers and determinants of strain dynamics following fecal microbiota transplantation. Nat. Med..

[30] Ding S., Jiang H., Fang J., Liu G. (2021). Regulatory Effect of Resveratrol on Inflammation Induced by Lipopolysaccharides via Reprograming Intestinal Microbes and Ameliorating Serum Metabolism Profiles. Front. Immunol..

[31] Cho J.-Y., Kang D.W., Ma X., Ahn S.-H., Krausz K.W., Luecke H., Idle J.R., Gonzalez F.J. (2009). Metabolomics reveals a novel vitamin E metabolite and attenuated vitamin E metabolism upon PXR activation. J. Lipid Res..

[32] Bae M., Ahmed K., Yim J.-E. (2022). Beneficial Effects of Taurine on Metabolic Parameters in Animals and Humans. J. Obes. Metab. Syndr..

[33] Devaraj S., Leonard S., Traber M.G., Jialal I. (2008). Gamma-tocopherol supplementation alone and in combination with alpha-tocopherol alters biomarkers of oxidative stress and inflammation in subjects with metabolic syndrome. Free Radic. Biol. Med..

[34] Pascale A., Marchesi N., Govoni S., Coppola A., Gazzaruso C. (2019). The role of gut microbiota in obesity, diabetes mellitus, and effect of metformin: new insights into old diseases. Curr. Opin. Pharmacol..

[35] American Diabetes Association (2020). 9. Pharmacologic approaches to glycemic treatment: Standards of medical care in diabetesd—2020. Diabetes Care.

[36] Brown M.D., Korytkowski M.T., Zmuda J.M., McCole S.D., Moore G.E., Hagberg J.M. (2000). Insulin sensitivity in postmenopausal women: independent and combined associations with hormone replacement, cardiovascular fitness, and body composition. Diabetes Care.

[37] Blum A. (2018). Freestyle Libre Glucose Monitoring System. Clin. Diabetes.

[38] Galindo R.J., Migdal A.L., Davis G.M., Urrutia M.A., Albury B., Zambrano C., Vellanki P., Pasquel F.J., Fayfman M., Peng L., Umpierrez G.E. (2020). Comparison of the FreeStyle Libre Pro Flash Continuous Glucose Monitoring (CGM) System and Point-of-Care Capillary Glucose Testing in Hospitalized Patients With Type 2 Diabetes Treated With Basal-Bolus Insulin Regimen. Diabetes Care.

[39] Wortelboer K., Koopen A.M., Herrema H., de Vos W.M., Nieuwdorp M., Kemper E.M. (2022). From fecal microbiota transplantation toward next-generation beneficial microbes: The case of Anaerobutyricum soehngenii. Front. Med..

[40] (2015). Milk and milk products — Starter cultures, probiotics and fermented products — Quantification of lactic acid bacteria by flow cytometry.

[41] Dalla Man C., Campioni M., Polonsky K.S., Basu R., Rizza R.A., Toffolo G., Cobelli C. (2005). Two-hour seven-sample oral glucose tolerance test and meal protocol: minimal model assessment of beta-cell responsivity and insulin sensitivity in nondiabetic individuals. Diabetes.

[42] Attaye I., van der Vossen E.W.J., Mendes Bastos D.N., Nieuwdorp M., Levin E. (2022).

[43] Hoogerland J.A., Lei Y., Wolters J.C., de Boer J.F., Bos T., Bleeker A., Mulder N.L., van Dijk T.H., Kuivenhoven J.A., Rajas F. (2019). Glucose-6-Phosphate Regulates Hepatic Bile Acid Synthesis in Mice. Hepatology.

[44] Chen L., van den Munckhof I.C.L., Schraa K., ter Horst R., Koehorst M., van Faassen M., van der Ley C., Doestzada M., Zhernakova D.V., Kurilshikov A. (2020). Genetic and Microbial Associations to Plasma and Fecal Bile Acids in Obesity Relate to Plasma Lipids and Liver Fat Content. Cell Rep..

[45] Wang D., Doestzada M., Chen L., Andreu-Sánchez S., van den Munckhof I.C.L., Augustijn H.E., Koehorst M., Ruiz-Moreno A.J., Bloks V.W., Riksen N.P. (2021). Characterization of gut microbial structural variations as determinants of human bile acid metabolism. Cell Host Microbe.

[46] Out C., Patankar J.V., Doktorova M., Boesjes M., Bos T., de Boer S., Havinga R., Wolters H., Boverhof R., van Dijk T.H. (2015). Gut microbiota inhibit Asbt-dependent intestinal bile acid reabsorption via Gata4. J. Hepatol..

[47] Koh A., Molinaro A., Ståhlman M., Khan M.T., Schmidt C., Mannerås-Holm L., Wu H., Carreras A., Jeong H., Olofsson L.E. (2018). Microbially Produced Imidazole Propionate Impairs Insulin Signaling through mTORC1. Cell.

[48] Koopen A.M., de Clercq N.C., Warmbrunn M.V., Herrema H., Davids M., de Groot P.F., Kootte R.S., Bouter K.E.C., Nieuwdorp M., Groen A.K., Prodan A. (2020). Plasma Metabolites Related to Peripheral and Hepatic Insulin Sensitivity Are Not Directly Linked to Gut Microbiota Composition. Nutrients.

[49] Blanco-Miguez A., Beghini F., Cumbo F., Mciver L.J., Thompson K.N., Zolfo M., Manghi P., Dubois L., Huang K.D., Thomas A.M. (2023). Extending and improving metagenomic taxonomic profiling with uncharacterized species with MetaPhlAn 4. Nat. Biotechnol..

[50] Emiola A., Oh J. (2018). High throughput *in situ* metagenomic measurement of bacterial replication at ultra-low sequencing coverage. Nat. Commun..

