# Supplementary material for: Oral Anaerobutyricum soehngenii augments glycemic control in type 2 diabetes
Source: iScience. 2024 Jul 5;27(8):110455. doi: 10.1016/j.isci.2024.110455 (PMC11321313; doi:10.1016/j.isci.2024.110455)
Supplement: Document S1. Figures S1–S9, Tables S1–S4, and Data S1 and S2 [file mmc1.pdf]

iScience, Volume *27*

Supplemental information

Oral *Anaerobutyricum soehngenii* augments glycemic control in type 2 diabetes

Ilias Attaye, Julia J. Witjes, Annefleur M. Koopen, Eduard W.J. van der Vossen, Diona Zwirs, Koen Wortelboer, Didier Collard, Elles Marleen Kemper, Maaike Winkelmeijer, Jens J. Holst, Stanley L. Hazen, Folkert Kuipers, Erik S.G. Stroes, Albert K. Groen, Willem M. de Vos, Max Nieuwdorp, and Hilde Herrema

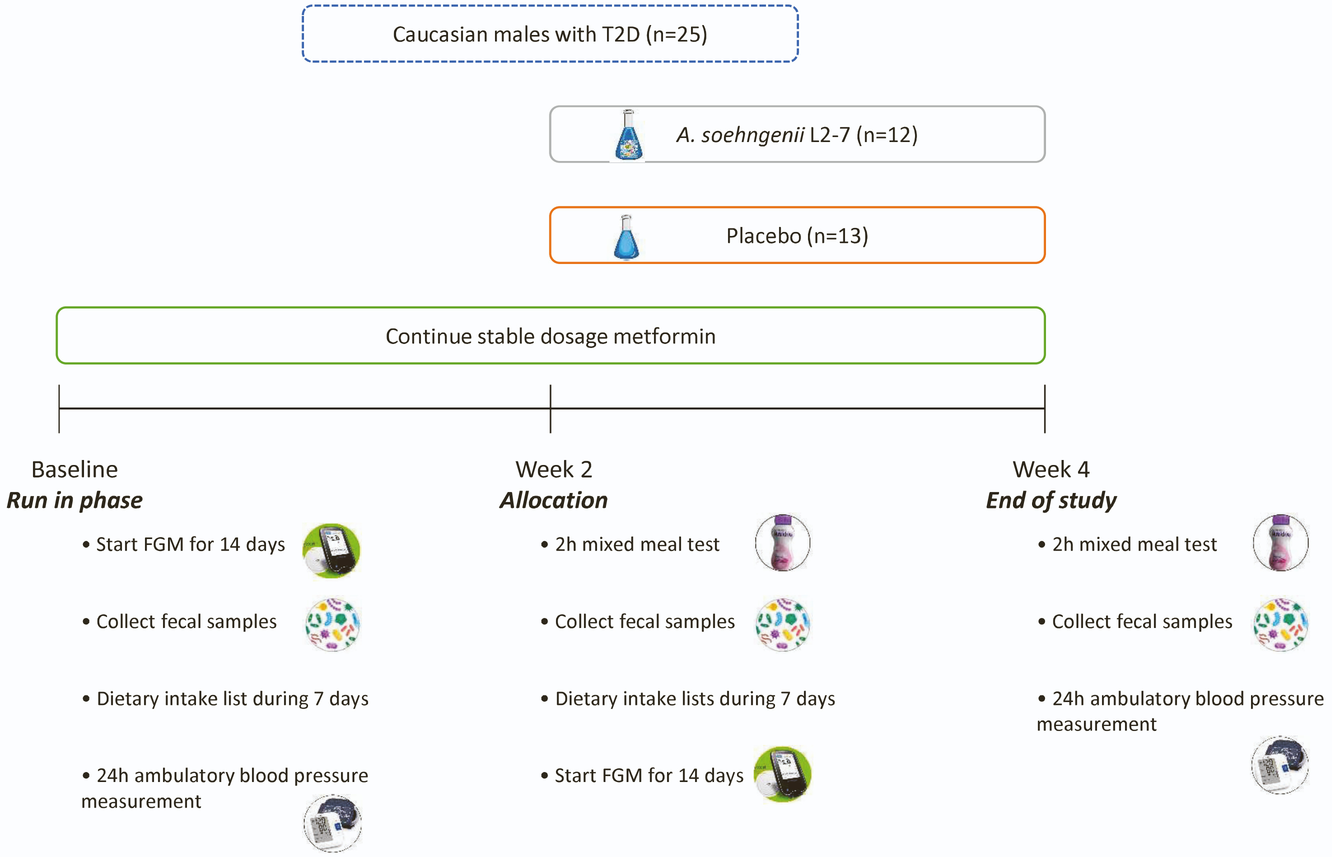

Suppl. Figure 1: Study design. Related to figure 1.

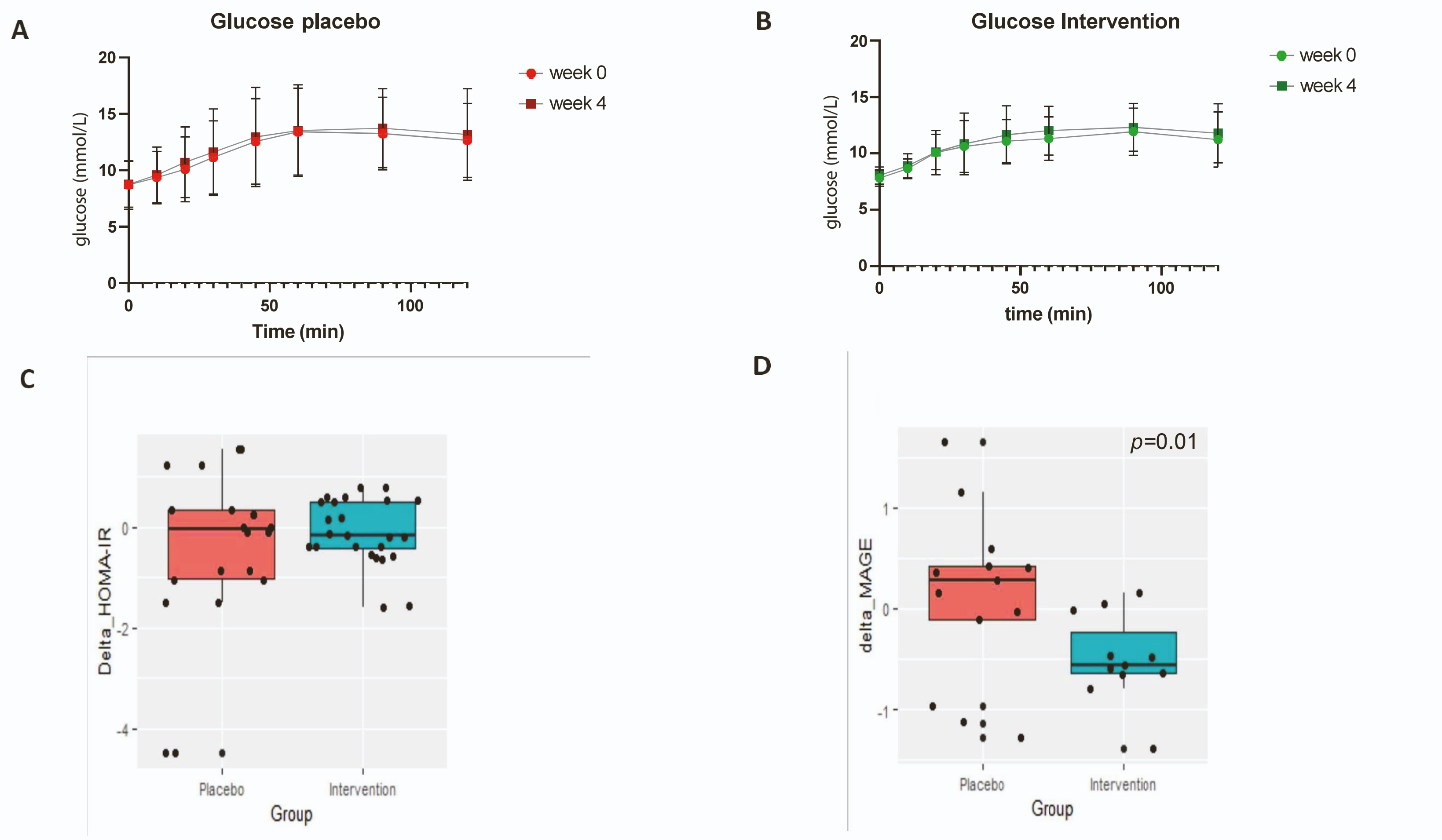

Suppl. Figure 2 :Effect of 14 days placebo (A) and A. soehngenii (B) supplementation on glucose levels following a 2-hour mixed-meal test, HOMA-IR (ns C), and mean amplitude of glycaemic excursions (MAGE) (D), Related to figure 1.

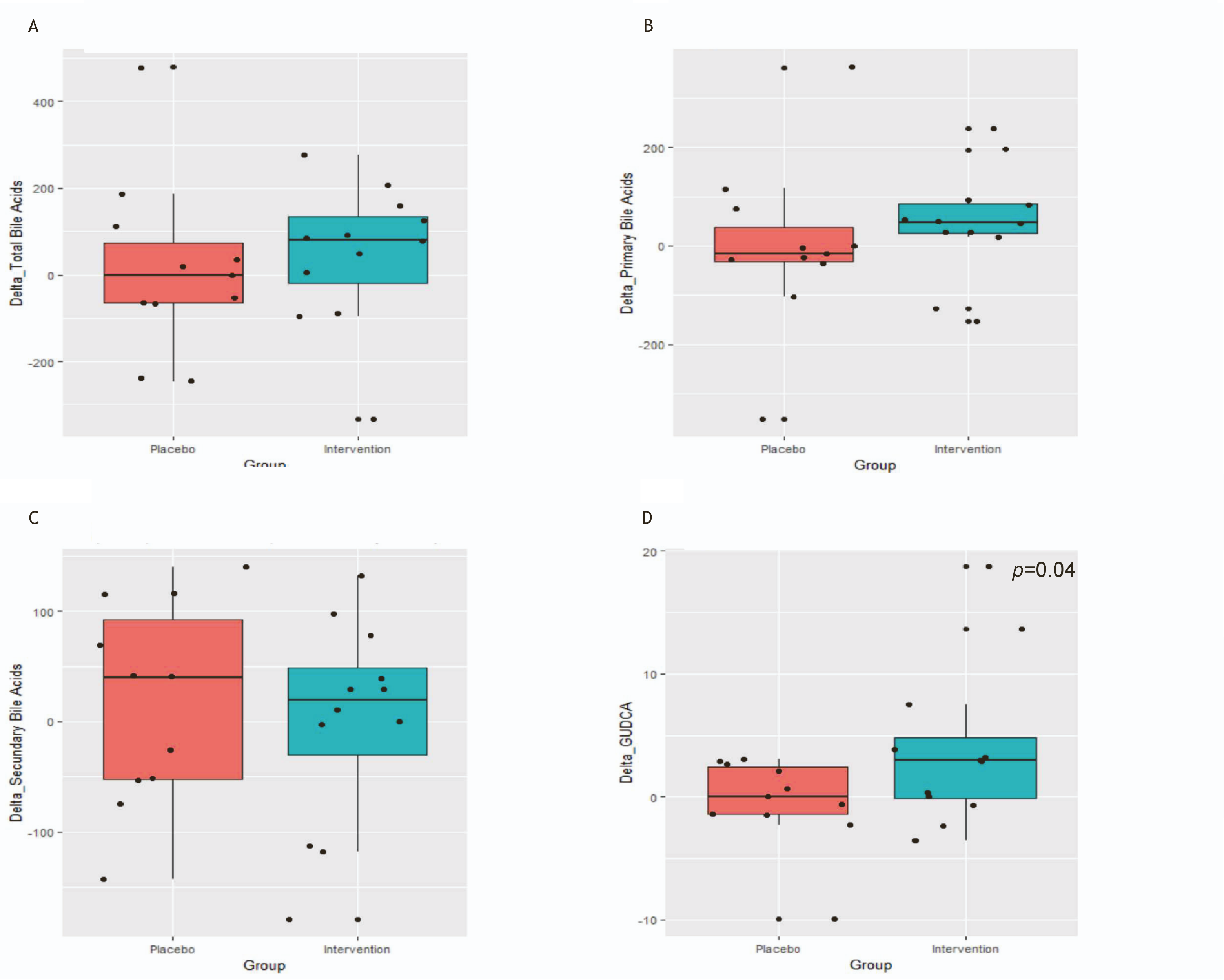

Suppl. Figure 3 :Effect of 14 days A. soehngenii supplementation on delta levels of plasma total bile acid including all subjects randomized (A), primary bile acids (B), secondary bile acids (C) and Glycoursodeoxycholic acid (GUDCA) (D). Related to figure 2.

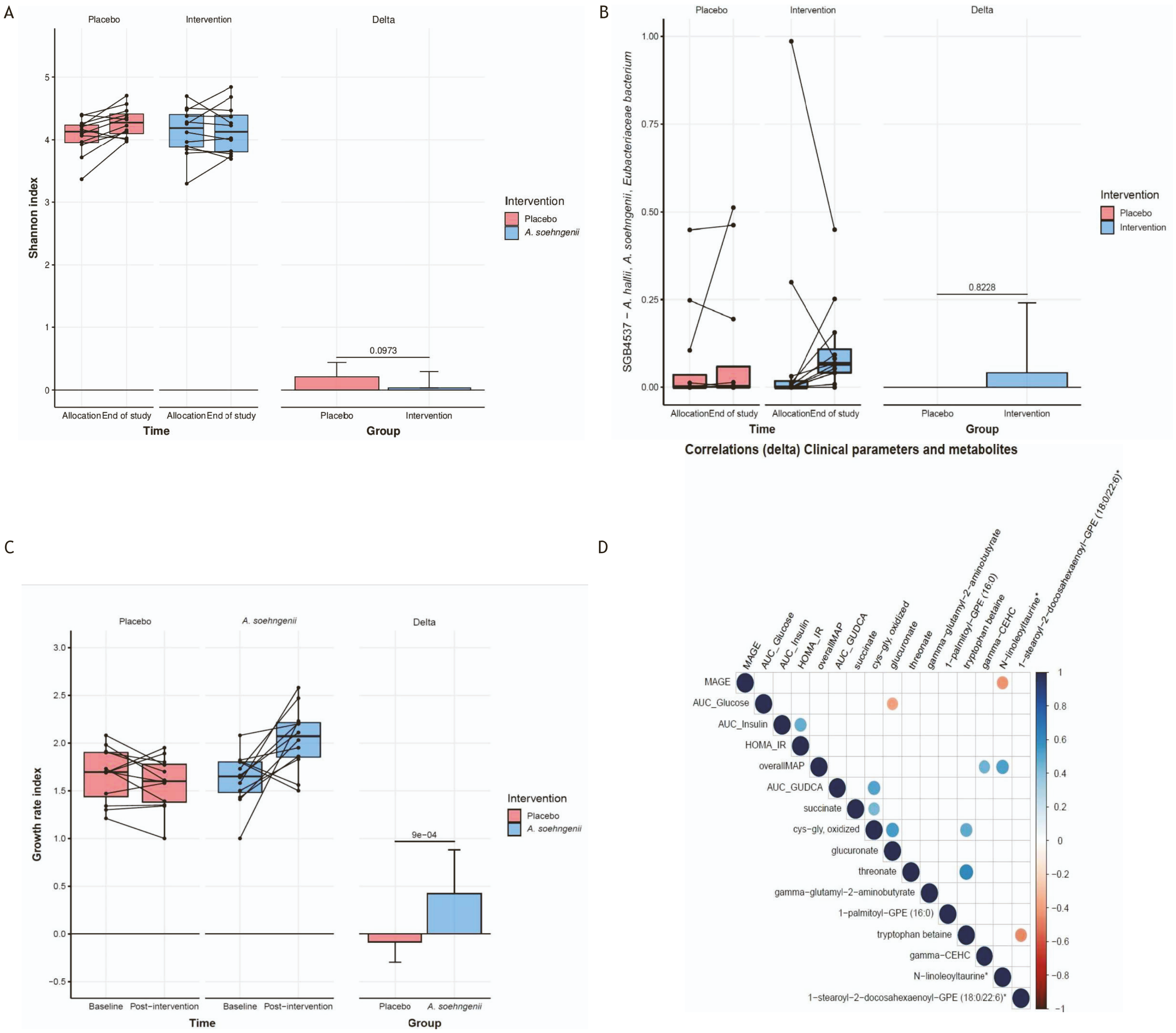

Suppl. Figure 4 :Effect of 14 days A. soehngenii supplementation on (A) Shannon index, (B) Fecal microbiota composition at baseline and end of the intervention, (C) Replication activity of the A. soehngenii between the placebo- and intervention group over time with the delta values (mean) and (D) Delta spearman correlations within and between metabolites and clinical parameters. Related to figure 3.

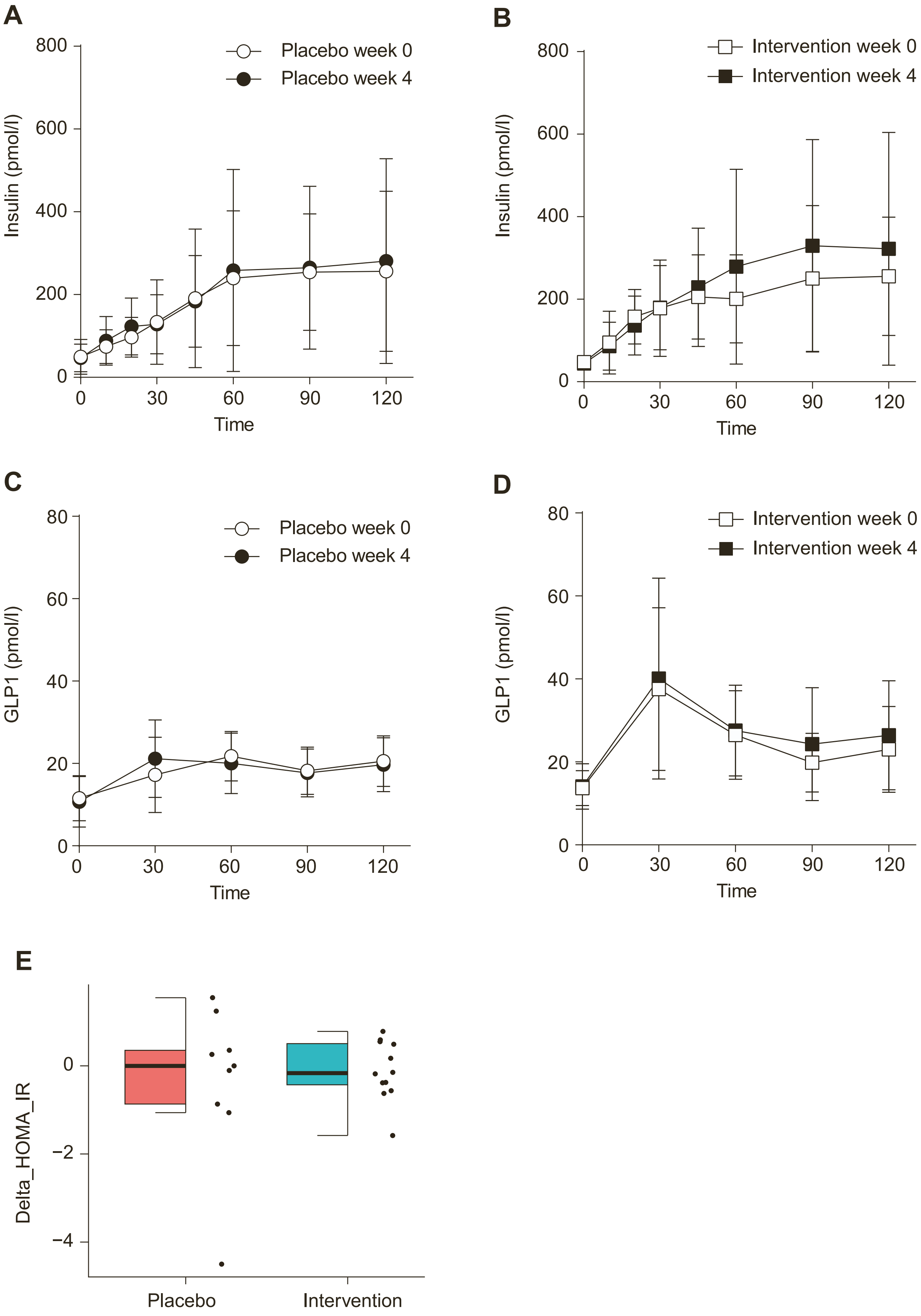

Suppl. Figure 5 :Effect of 14 days placebo (A) and A. soehngenii (B) supplementation on insulin levels, placebo (C) and A. soehngenii (D) supplementation on glucagon-like peptide 1 (GLP-1) levels and (E) Effect of placebo or A. soehngenii on ΔHOMA-IR following 14 days of supplementation Related to figure 1.

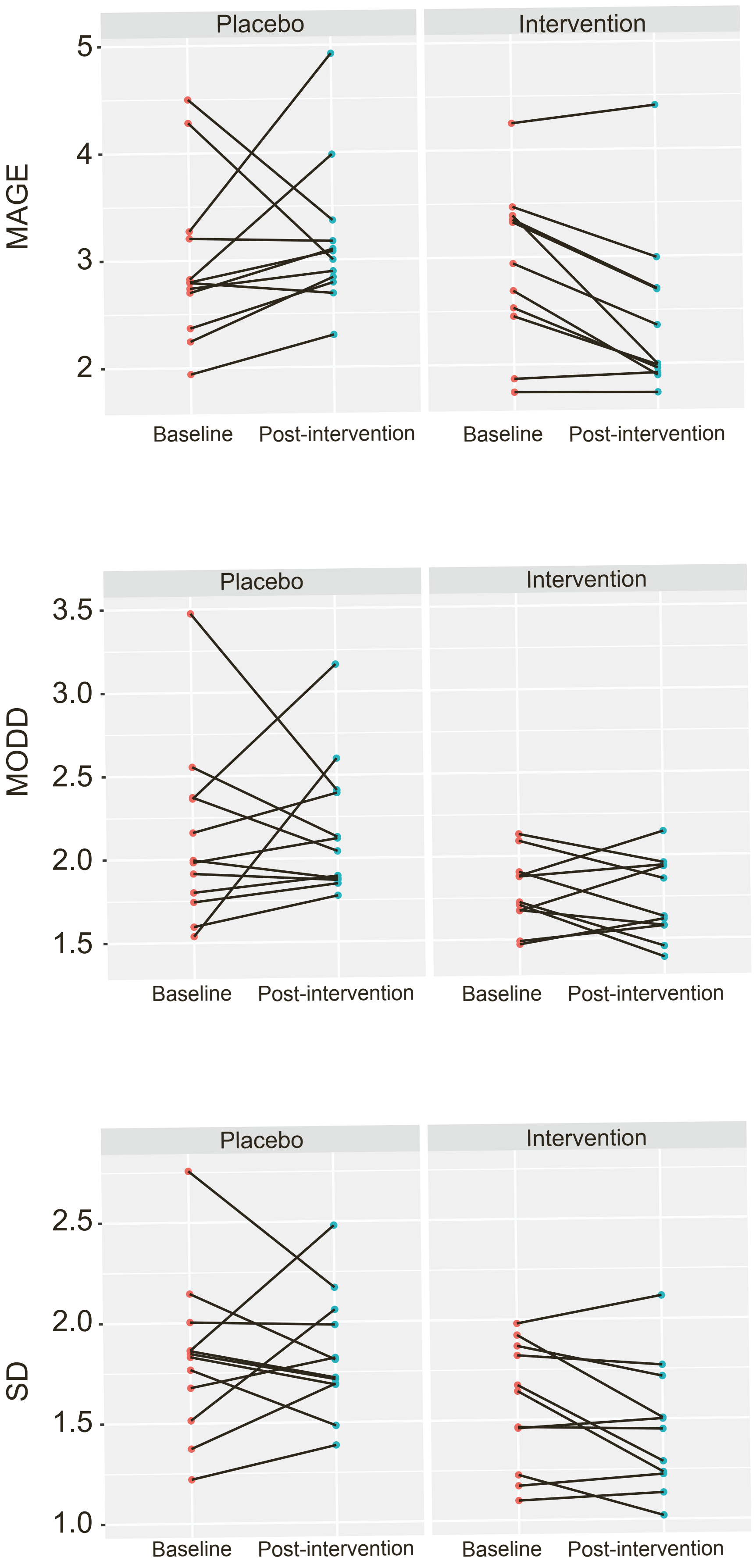

Suppl. Figure 6: Individual responses following placebo or A. soehngenii supplementation for 14 days on Mean Amplitude of Glucose Excursions (MAGE), Mean Of Daily Differences (MODD) and Standerd Deviation (SD), Related to figure 1.

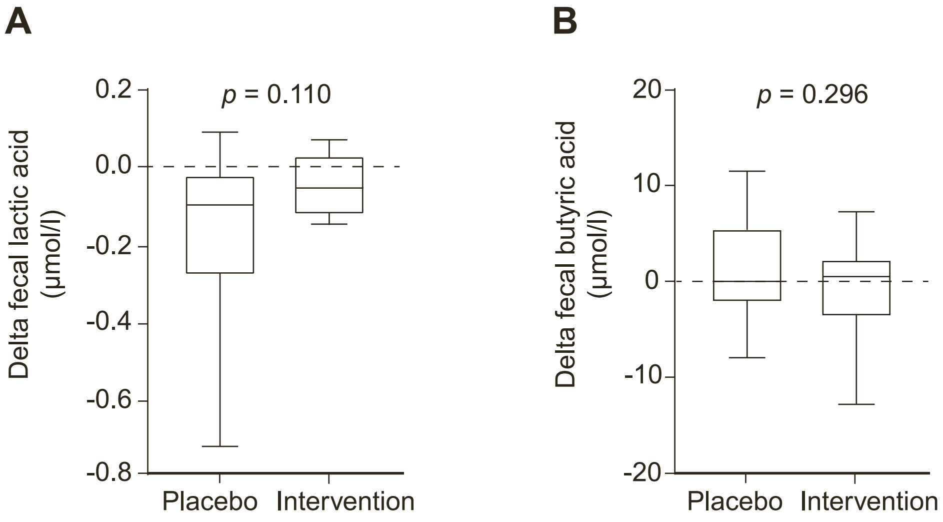

Suppl. Figure 7: Fecal lactic acid and butyric acid levels expressed as delta concentration within the placebo and intervention group Related to figure 1.

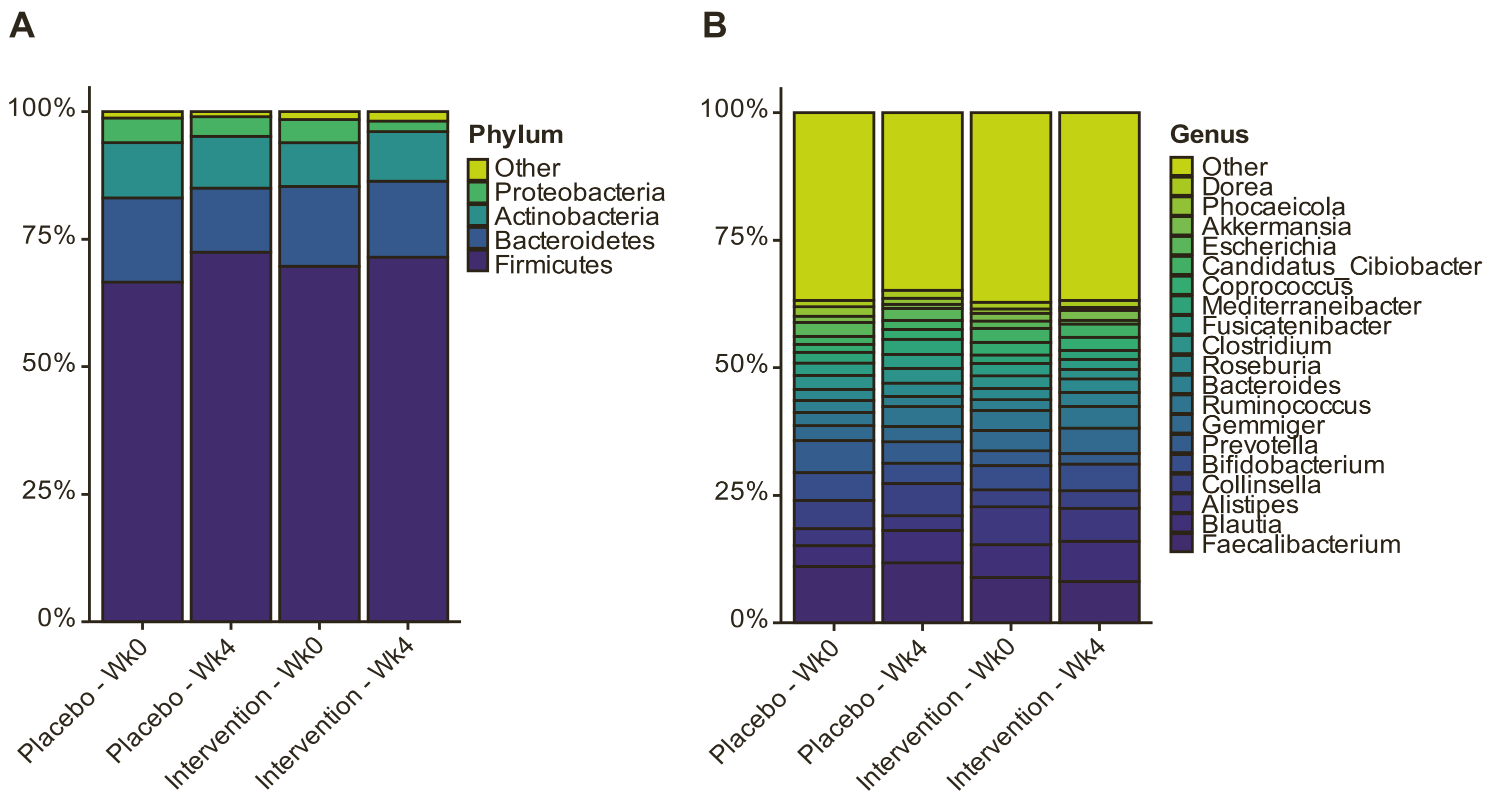

Suppl. Figure 8: Tax bar plots of fecal bacterial groups at Phylum (A) and Genus (B) level in the placebo group (week 0 and week 4) and in the intervention group (week 0 and week 4). Related to figure 3.

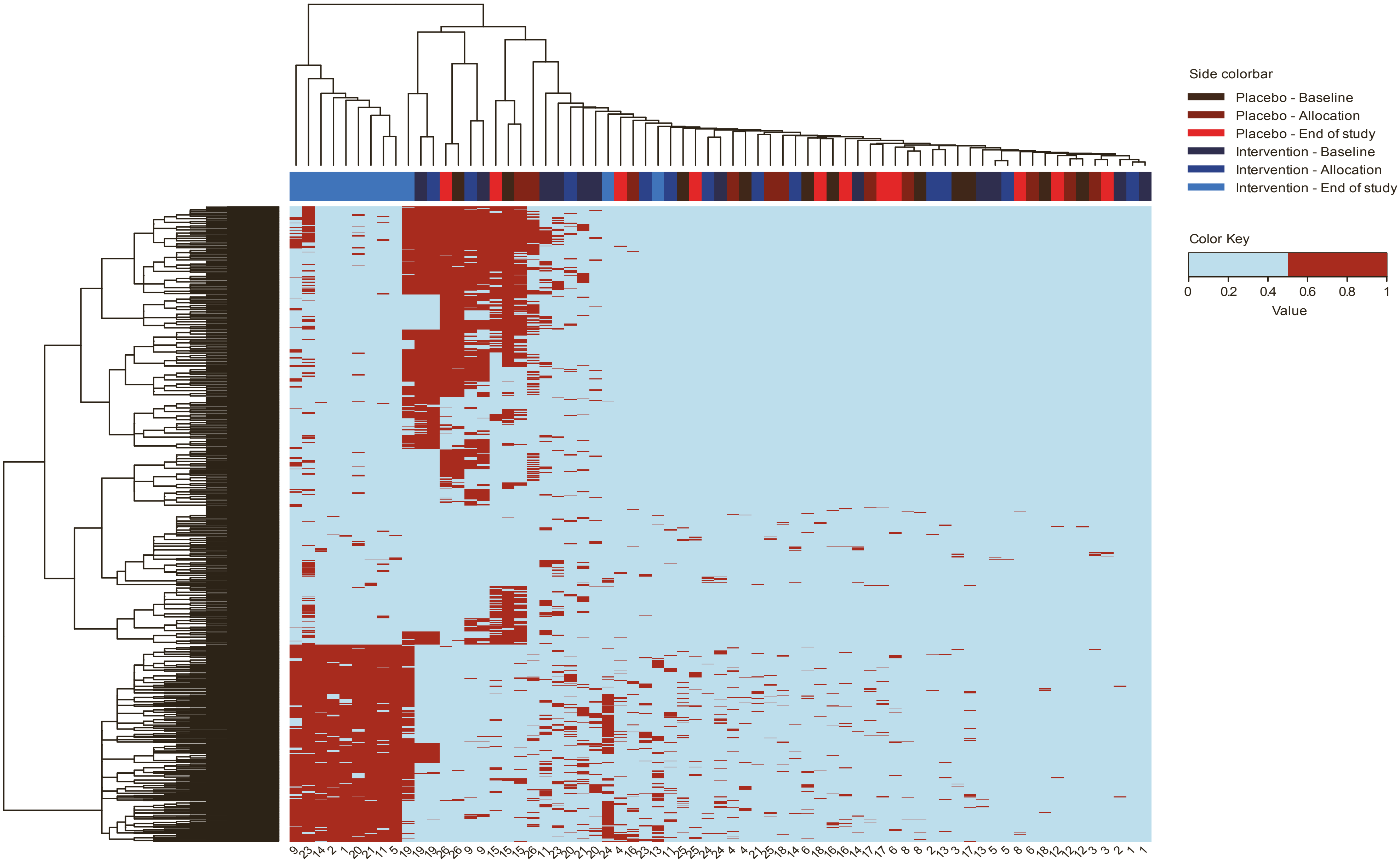

Suppl. Figure 9 Shows filtered dichotomized clustered SNP profiles of different subjects at different time points. Related to figure 3.

Suppl Table 1: Baseline characteristics of the EDM2 study. Related to figure 1.

	Placebo (n = 12)	Intervention (n = 12)	*p*-value
Male sex (%)	100	100	
Age (y)	60.6 (5.7)	60.8 (6.2)	0.95
BMI (kg/m2)	27.2 (2.6)	27.7 (4.3)	0.73
Weight (kg)	86.41 (13.03)	91.35 (17.30)	0.44
Systolic blood pressure (mmHg)	133.50 (13.96)	141.00 (17.68)	0.26
Diastolic blood pressure	86.42 (5.11)	82.58 (8.45)	0.19
Fasting glucose (mmol/L)	8.47 (2.22)	7.83 (0.66)	0.35
Insulin (pmol/L)	45.00 (33.98)	45.75 (15.63)	0.95
HbA1c (%)	6.96 (1.01))	6.60 (0.49)	0.28
Total Cholesterol (mmol/L)	5.31 (0.65)	4.97 (0.85)	0.28
HDL (mmol/L)	1.32 (0.26)	1.30 (0.23)	0.86
LDL (mmol/L)	3.26 (0.59)	3.05 (0.63)	0.42
Triglycerides (mmol/L)	1.65 (1.06)	1.38 (0.54)	0.45
AST (U/L)	186.00 (379.80)	21.25 (3.44)	0.15
ALT (U/L)	29.50 (14.55)	23.50 (9.90)	0.25
AP (U/L)	71.17 (24.86)	66.33 (17.45)	0.59
yGT (U/L)	36.50 (29.86)	25.67 (20.03)	0.31
CRP (mg/mL)	1.98 (3.48)	1.10 (0.68)	0.396
Leucocytes (10^9/L)	5.97 (1.22)	5.86 (1.33)	0.84
Caloric intake (kcal/day)	1682.05 (351.67)	1727.95 (383.90)	0.78
Fat intake (g/day)	69.40 (21.98)	73.06 (21.22)	0.70
Carbohydrates intake (g/day)	153.46 (59.86)	150.22 (53.99)	0.90
Protein intake (g/day)	85.88 (39.95)	89.32 (21.55)	0.81
Fiber intake (g/day)	18.23 (5.03)	19.85 (6.75)	0.55

Suppl Table 2: Plasma short-chain fatty acid AUC over 120 min (mean (SD)). Related to figure 1.

**Baseline**	Placebo (n=12)	Intervention (n=12)	**End of intervention**	Placebo (n=12)	Intervention (n=12)	**p-value**
SCFA_acetic_AUC	23494.82 (2462.42)	25057.38 (5631.50)	SCFA_acetic_AUC	23307.50 (4044.07)	25539.27 (3863.17)	0.754
SCFA_Butyric_AUC	1688.12 (107.43)	1855.53 (604.98)	SCFA_Butyric_AUC	1997.70 (313.63)	2089.82 (176.85)	0.716
SCFA_Proprionic_AUC	3181.20 (260.13)	3368.52 (895.78)	SCFA_Proprionic_AUC	3503.62 (577.02)	3625.95 (251.93)	0.846
SCFA_Isovaleric_AUC	225.28 (31.00)	251.88 (25.91)	SCFA_Isovaleric_AUC	204.83 (42.90)	228.43 (35.12)	0.819
SCFA_Lactic_AUC	216732.30 (94759.21)	185878.25 (78406.90)	SCFA_Lactic_AUC	201601.15 (91917.90)	176047.38 (39557.45)	0.854
SCFA_Succinic_AUC	2213.20 (997.30)	2241.75 (839.27)	SCFA_Succinic_AUC	2107.00 (594.24)	1844.55 (581.08)	0.466
SCFA_total_AUC	247534.92 (94962.18)	218653.30 (84026.25)	SCFA_total_AUC	232721.80 (92122.29)	209375.40 (39801.44)	0.859

Suppl Table 3: 24-hours-fecal bile acids levels (mean (SD)). Related to figure 2.

**Baseline**	Placebo (n=12)	Intervention(n=12)	**End of Intervention**	Placebo (n=12)	Intervention(n=12)	p-value
UDCA_AUC	5.70 (3.15)	4.90 (3.08)	UDCA_AUC	7.10 (4.53)	6.31 (3.27)	0.623
CA_AUC	20.51 (42.65)	5.42 (1.69)	CA_AUC	18.01 (22.33)	26.51 (57.92)	0.283
GUDCA_AUC	9.76 (6.35)	9.89 (5.40)	GUDCA_AUC	9.40 (4.81)	13.71 (8.63)	0.081
GCA_AUC	48.37 (31.74)	70.34 (61.63)	GCA_AUC	47.85 (38.29)	68.59 (45.07)	0.958
TUDCA_AUC	1.49 (0.57)	2.04 (1.03)	TUDCA_AUC	1.53 (0.58)	2.34 (1.15)	0.579
TCA_AUC	11.24 (7.83)	20.58 (18.01)	TCA_AUC	11.47 (9.06)	16.69 (9.20)	0.437
CDCA_AUC	35.15 (36.11)	16.34 (16.08)	CDCA_AUC	41.21 (45.79)	34.19 (34.82)	0.105
DCA_AUC	74.51 (36.37)	59.58 (32.74)	DCA_AUC	72.63 (37.67)	77.22 (48.58)	0.321
GCDCA_AUC	149.35 (81.93)	154.86 (95.86)	GCDCA_AUC	152.55 (105.88)	163.45 (84.77)	0.743
GDCA_AUC	94.27 (83.99)	131.75 (115.80)	GDCA_AUC	69.74 (47.02)	127.08 (116.28)	0.709
TCDCA_AUC	21.60 (15.28)	34.55 (23.74)	TCDCA_AUC	24.53 (21.39)	38.21 (27.71)	0.821
TDCA_AUC	18.65 (18.24)	29.70 (21.42)	TDCA_AUC	14.54 (8.91)	25.84 (12.82)	0.919
TLCA3S_AUC	14.36 (14.30)	19.94 (9.66)	TLCA3S_AUC	13.20 (9.08)	17.25 (7.41)	0.812
LCA_AUC	5.48 (1.32)	5.80 (2.97)	LCA_AUC	4.95 (1.23)	5.42 (2.63)	0.93
TLCA_AUC	2.59 (1.37)	4.09 (1.24)	TLCA_AUC	2.77 (1.10)	3.76 (1.22)	0.172
GLCA_AUC	9.59 (5.90)	12.64 (11.03)	GLCA_AUC	8.85 (4.80)	12.32 (10.18)	0.468
LCA3S_AUC	1.87 (1.02)	1.81 (1.02)	LCA3S_AUC	2.48 (1.38)	2.91 (1.52)	0.463
GLCA3S_AUC	55.72 (33.99)	67.88 (57.35)	GLCA3S_AUC	47.09 (22.93)	57.41 (38.57)	0.766
Total_AUC	580.43 (308.42)	651.72 (339.46)	Total_AUC	549.44 (269.00)	698.66 (299.71)	0.394
Prim_AUC	286.15 (182.76)	301.85 (177.51)	Prim_AUC	295.53 (215.63)	347.36 (170.35)	0.364
Secon_AUC	294.41 (173.49)	349.89 (195.83)	Secon_AUC	254.00 (91.01)	351.34 (187.95)	0.608

Suppl Table 4: Plasma bile acid AUC over 120 min (mean (SD)).. Related to figure 2.

**Baseline**	Placebo (n=11)	Intervention (n=11)	**End of intervention**	Placebo (n=11)	Intervention (n=11)	**p-value**
UDCA	0.16 (0.28)	0.06 (0.11)	UDCA	0.08 (0.14)	0.09 (0.19)	0.148
CA	0.59 (1.19)	0.33 (0.84)	CA	0.15 (0.19)	0.23 (0.29)	0.404
GUDCA	0.01 (0.01)	0.01 (0.00)	GUDCA	0.01 (0.01)	0.01 (0.00)	0.685
GCA	0.07 (0.06)	0.03 (0.04)	GCA	0.10 (0.15)	0.05 (0.06)	0.877
TUDCA	0.00 (0.00)	0.00 (0.00)	TUDCA	0.00 (0.00)	0.00 (0.00)	0.493
TCA	0.02 (0.02)	0.03 (0.03)	TCA	0.03 (0.03)	0.04 (0.04)	0.513
CDCA	0.60 (0.89)	0.31 (0.43)	CDCA	0.24 (0.16)	0.27 (0.17)	0.258
DCA	10.23 (6.57)	7.47 (4.38)	DCA	11.03 (6.73)	7.25 (4.29)	0.445
GCDCA	0.09 (0.05)	0.05 (0.02)	GCDCA	0.10 (0.08)	0.06 (0.03)	0.939
GDCA	0.10 (0.07)	0.08 (0.05)	GDCA	0.09 (0.07)	0.08 (0.06)	0.639
TCDCA	0.03 (0.02)	0.03 (0.02)	TCDCA	0.04 (0.03)	0.04 (0.02)	0.429
TDCA	0.02 (0.02)	0.03 (0.02)	TDCA	0.03 (0.02)	0.03 (0.02)	0.915
LCA	4.27 (1.76)	3.50 (1.37)	LCA	5.51 (2.22)	3.27 (1.60)	**<0.001**
TLCA	0.01 (0.00)	0.01 (0.01)	TLCA	0.01 (0.01)	0.01 (0.01)	0.068
GLCA	0.01 (0.00)	0.02 (0.01)	GLCA	0.01 (0.00)	0.02 (0.01)	0.585
Total	16.21 (9.95)	11.96 (6.47)	Total	17.42 (9.25)	11.45 (5.95)	0.327
sum_Coprostanol_cholesterol	50.61 (21.08)	49.68 (22.54)	sum_Coprostanol_cholesterol	56.57 (19.89)	57.80 (22.51)	0.844

Suppl. Data S1: Study protocol. Related to figure 1.

The EMDM2 trial

Effect of oral Eubacterium Hallii on postprandial glucose metabolism in males with type 2 diabetes treated with metformin

Department of Internal and Vascular Medicine, AMC

Department of Microbiology, WUR

PROTOCOL TITLE: Effect of oral Eubacterium Hallii on postprandial glucose metabolism in males with type 2 diabetes treated with metformin

**Protocol ID**	EMDM2 trial
**Short title**	E hallii with metformin in type 2 diabetes
**Manuscript title**	Oral treatment with Anaerobutyricum soehngenii augments glycemic control in male individuals with type 2 diabetes treated with metformin: a randomized double-blind placebo-controlled single center study
**Trial registration**	WHO International Dutch Clinical Trial Register: https://clinicaltrialregister.nl/nl/trial/28536 ; registration number NL7121
**Institutional Review Board (IRB) approval**	Current protocol was approved by the IRB of Amsterdam UMC, location AMC
**Corresponding author**	Dr Hilde Herrema (mail: h.j.herrema@amsterdamumc.nl ; phone :+31 020 566 6118)

TABLE OF CONTENTS

1. INTRODUCTION AND RATIONALE 7
2. OBJECTIVES 12
3. STUDY DESIGN 13
4. STUDY POPULATION 15
4.1 Population 15
5. TREATMENT OF SUBJECTS 17
5.1 Investigational product/treatment 17
5.2 Use of co-intervention 17
6. METHODS 17
6.1 Study parameters/endpoints 17
6.1.1 Main study parameter/endpoint 18
6.1.2 Secondary study parameters/endpoints 18
6.2 Randomization, blinding and treatment allocation 18
6.3 Study procedures 18
6.3.1 Effect on postprandial glucose excursions 18
6.4 Withdrawal of individual subjects 19
6.5 Replacement of individual subjects after withdrawal 19
6.6 Follow-up of subjects withdrawn from treatment 19
7. SAFETY REPORTING 20
7.1 Temporary halt for reasons of subject safety 20
7.2 AEs, SAEs and SUSARs 20
7.2.1 Adverse events (AEs) 20
7.2.2 Serious adverse events (SAEs) 20
7.3 Follow-up of adverse events 21
8. STATISTICAL ANALYSIS 22
9. ETHICAL CONSIDERATIONS 23
9.1 Regulation statement 23
9.2 Recruitment and consent 23
9.3 Compensation for injury 23
9.4 Incentives 23
10. ADMINISTRATIVE ASPECTS, MONITORING AND PUBLICATION 24
10.1 Handling and storage of data and documents 24
10.2 Amendments 24
10.3 Annual progress report 24
10.4 End of study report 25
10.5 Public disclosure and publication policy 25
11. REFERENCES 26

SUMMARY

Objective: to investigate the effect of 14 days once daily oral Eubacterium hallii (E. hallii) treatment on postprandial glucose levels in relation to SCFA levels in feces in patients with type 2 diabetes treated with metformin.

Study design: randomized, double-blind, placebo-controlled single center study

Study Population: 24 caucasian males with type 2 diabetes treated with stable metformin monotherapy

Intervention: Subjects will be given oral 10 ml E. hallii suspension with a total concentration of 10e9 cells/ml in 10% glycerol or 10ml 10 % glycerol only (both produced by NIZO and stored at Dept of Clinical Pharmacy AMC) once daily during 14 days. Standardized mixed meal test and 14 days wearing of a glucose sensor (CGM Freestyle libre) to measure effect on (postprandial) glucose levels.

Outcome measures: The primary endpoint is the effect of E. hallii versus placebo on (postprandial) glucose excursions as determined by a wearable CGM glucose sensor during 14 days after the start of the intervention. Secondary endpoints are changes in plasma metabolites and glucose/lipids upon standardized meal in relation to changes of E. hallii and other microbiota as well as plasma/fecal SCFA in fecal samples. Also Daily dietary intake will be monitored during the course of the study by https://mijn.voedingscentrum.nl/nl/eetmeter.

Sample Size: We hypothesize that a peak–difference in postprandial glucose excursions using CGM is seen between type 2 diabetes patients using placebo + metformin (13.0 mmol/l) vs E hallii + metformine (10.0 mmol/l) with a SD of 2.5 mmol/l; using unpaired t-test with 0.05 two-sided significance levels and 80% power, we will need 12 subjects per group to detect a significant difference. Of note, all power calculations were performed with an online power calculation (www.biomath.info/power/). Based on the data retrieved from this intervention study, we will apply the prediction algorithm by Zeevi et al for validation[1]. We will include 24 males with type 2 diabetes treated with metformin. All will receive 2 weeks treatment with either E. hallii or 10% glycerol placebo.

Nature and extent of the burden and risks associated with participation, benefit and group relatedness: The total duration of this study is 4 weeks and participants will visit the AMC four (screening, run in, randomization and end of study visit) times. All participants are

required to fill out food diaries three days per week and are required to collect feces at baseline, week 2 and week 4 of the study. Furthermore, subjects will undergo a mixed meal test (MMT) before and after the intervention with blood sampling during 2 hours from a placed venflon. Afterwards, subjects are provided with a disposable continuous glucose sensor (FreeStyle Libre)[1], which will be worn for 14 days before and 14 days after the intervention to monitor (postprandial) glucose excursions both short term and long term, with this Free Style glucose sensor no additional blood glucose measurements with finger pricks need to be done. Also, subjects receive an blood pressure monitor to ambulatory measure their blood pressure during 24 hours before and after treatment. In total subjects will spent approximately 6 hours in the AMC (screening visit, 2x 2 hours for the MMT plus CGM at baseline, randomization and after intervention) and we will collect 240 ml blood (at baseline, week 2 and week 4) in total.

1. INTRODUCTION AND RATIONALE

The prevalence of type 2 diabetes is expected to rise to 33% of the adult population in 2050[2]. The pathophysiology of this disease is complex, involving both environmental (dietary) and genetic factors affecting altered intestinal microbiota composition. The development of culture-independent approaches, using high-throughput metagenomic sequencing via 16S rRNA[3], has drastically increased the knowledge of the gut microbiome, now linking any disturbances in it, both in human and animal models, to the pathophysiology of metabolic diseases such as obesity and type 2 diabetes mellitus (T2DM)[4–6]. Transplantation of lean healthy microbiota in subjects with insulin resistance showed an significantly increased insulin sensitivity and an increased abundance of butyrate-producing bacteria in the gut[7]. In this pilot study we identified a specific increase in the butyrate-producer *Eubacterium hallii* in small intestinal biopsies of human obese and insulin resistant subjects upon lean donor fecal transplantation (see figure 1).

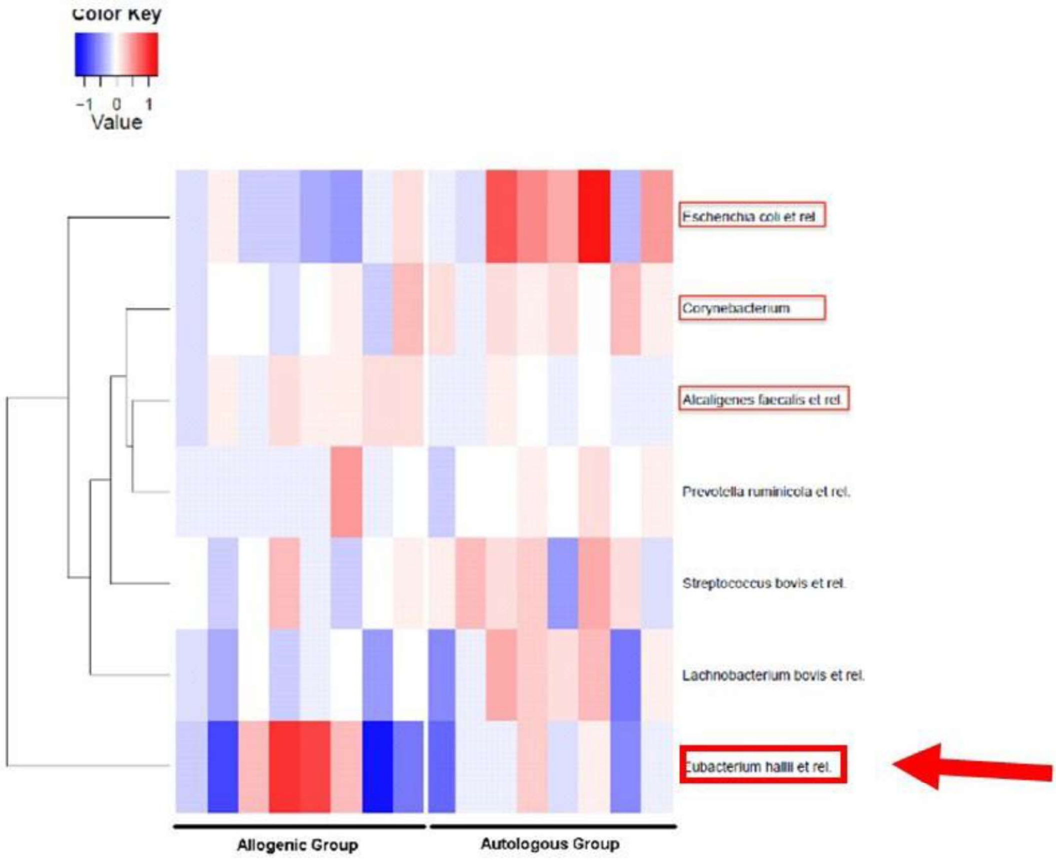

Figure 1. Increased Eubacterium Hallii levels in small intestinal biopsies of insulin resistant subjects 6 weeks after being treated with allogenic (lean donor) feces.

E. hallii is an anaerobic, Gram-positive, catalase-negative bacterium belonging to the clostridial cluster XIVa of the phylum Firmicutes and is present in both murine as human feces [8,9]. It is a butyrate-producing species, but in contrast to other well-known human

isolates such as Roseburia and Faecalibacterium spp. that produce butyrate from monosaccharides, E. hallii has the capacity to produce butyrate from lactate and acetate in an acid environment (pH 1-2) as found in the small intestine[9]. This makes it very plausible that this bacterial strain can effectively survive in low pH environments. Moreover, E. hallii has the capability to convert a potentially damaging acid (e.g. lactic acid) into other short chain fatty acid butyrate, which is known to exert beneficial effects on glucose metabolism[9].

Eubacterium hallii type strain L2-7 is available from the DSMZ (Deutsche Sammlung von Mikroorganismen und Zellkulturen) as DSM 17630. Its 16S rRNA has been sequenced and deposited at GenBank (see accession number AJ270490 – http://www.ncbi.nlm.nih.gov/nuccore/AJ270490).

We recently published an animal study in which we studied the effect of E. hallii treatment on metabolism in mice[10]. We found that increasing dosage of daily E. hallii treatment was safe and did not induce adverse effects. Moreover we observed a dose dependent effect of E. hallii on improved insulin sensitivity (ITT fig 2a) in correspondence with fecal E. hallii levels (fig 2b). Furthermore, we found significant changes in hepatic as well as small intestinal genes involved in bile acid and glucose metabolism (fig 2c) including a reduction in FXR expression, which is associated with improved insulin sensitivity and resulted in less hepatic triglycerides and obesity in mice[11].

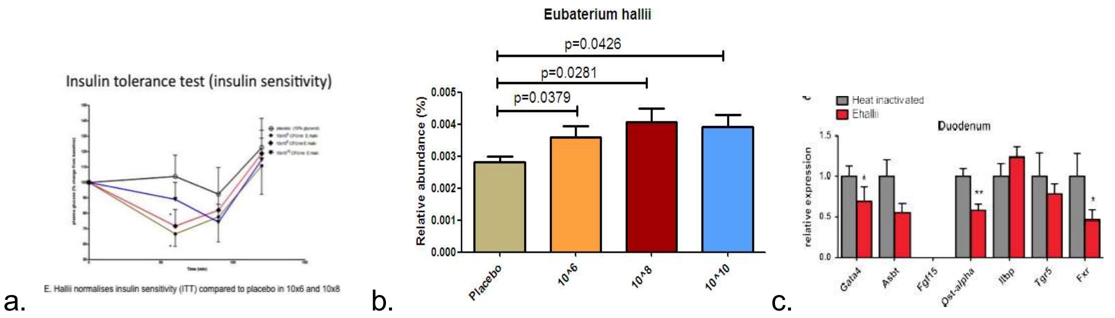

Figure 2. Results of dosefinding study of daily E.Hallii treatment on a) insulin sensitivity (as assessed by insulin tolerance test), b) concentrations of Eubacterium hallii in feces and c) small intestinal genes involved in bile acid metabolism upon 10E9 E hallii. FXR denotes farnesoid X receptor, a gene central in bile acid and glucose metabolism

In the recently finished DIME study (METC 2014_285) we studied safety and optimal dose of daily ingested oral E. hallii L2-7 strain for 4 weeks on insulin resistance/lipid metabolism in 27 male subjects with metabolic syndrome. Subjects were given increasing end concentration dosages of 10e5/day (low), 10e7/day (middle) and 10e9/day (high) E. hallii cells in 10 ml vials (dissolved in 10% glycerol) (n=9 subjects per group). No side effects were seen on either dosage during the treatment.

Although we saw no overall significant effect on changes in Rd (peripheral) insulin sensitivity per group, we did find a relation between increase in fecal levels of E. hallii upon treatment and a response in Rd (as defined by a clinical relevant insulin sensitivity increase Rd>10%); low dose: 2 out of 9, middle dose: 4 out of 9 and highest dose: 5 out of 9 responders (fig 3).

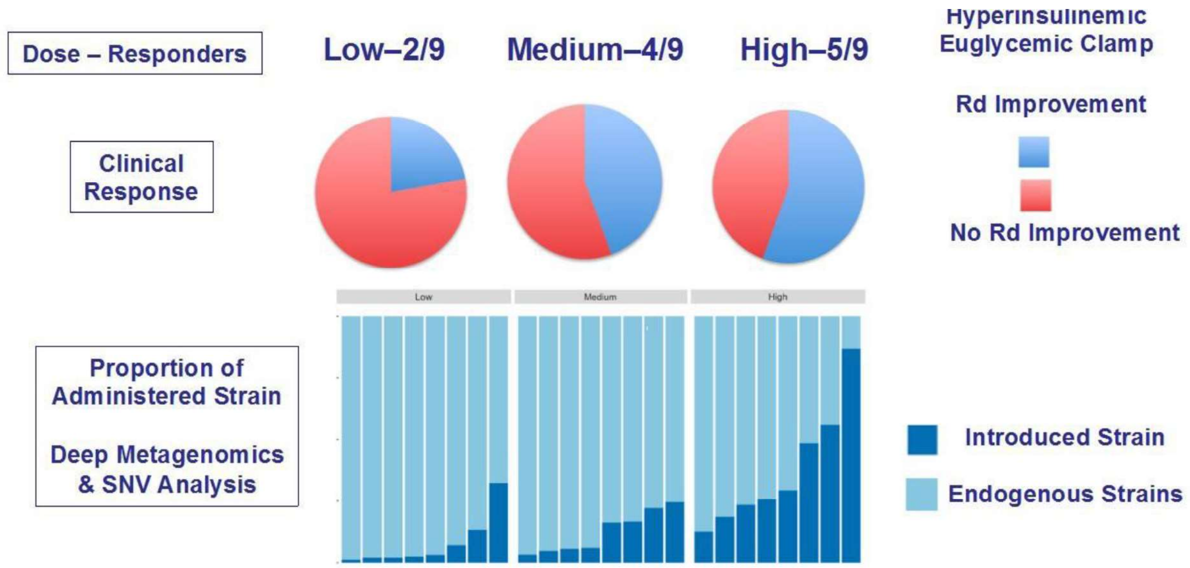

Figure 3. Amount of metabolic responders upon E halii treatment (upper panel) as well as dose dependent in increase of fecal E hallii L2-7 levels compared to endogenous strains (left panel: low, middle panel: middle dose and right panel: high dose).

In contrast with 16S sequencing (that showed no differences in E hallii concentrations), by using strain specific shotgun sequencing of the whole bacterial genome, we did find a significant correlation between E Hallii relative abundance and improvement in insulin sensitivity (Rd) with the largest effect in the highest dose group of 10e9/day (high) E. hallii once daily ($r = 0.4$, $p<0.05$) (Fig 4). Based on the fact that we administered daily E hallii in a drink, we thus postulate that in the group of responders, E. hallii is more likely to pass the stomach and thus exert beneficial effects/engraft in the (small) intestinal microbiota and improve metabolism.

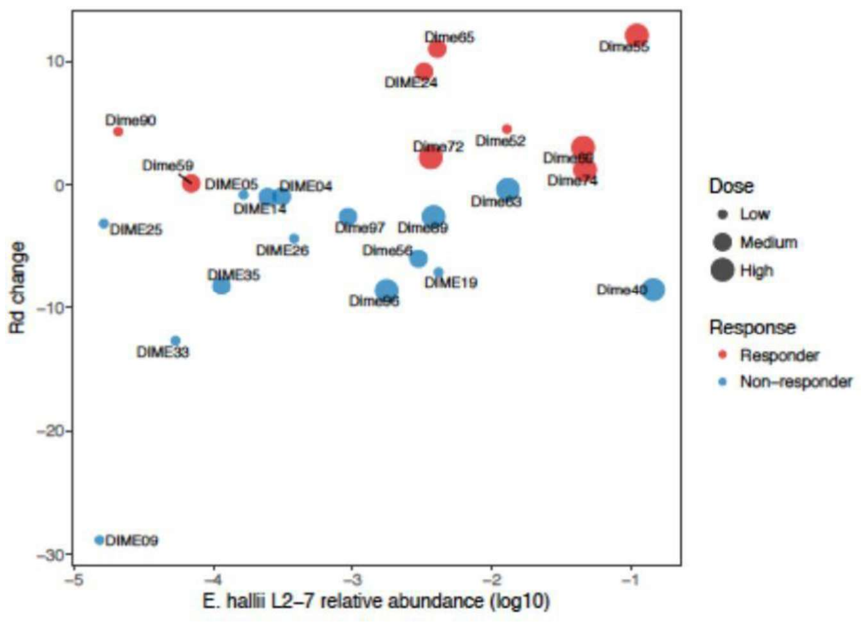

Figure 4. Linear correlation between Rd change (peripheral insulin sensitivity on y-axis0 and changes in fecal E halli L2-7 concentrations (as expressed by relative abundance on x-axis) in 27 metabolic syndrome subjects. A significant linear correlation was found between increase in Rd and increase in fecal E hallii levels (r=0.4, P<0.05) when pooling all groups.

In this regard, it is interesting to note that DM2 subjects on metformin treatment have increased levels of lactate in their feces[12]. Since E hallii uses intestinally produced lactate to produce butyrate that is thought to be the beneficial compound driving the effects on insulin sentivity, we hypothesize that adding E hallii to metformin treatment in subjects with DM2 may improve their glycemic control. This as the produced lactate by metformin treatment potentially could be converted by E hallii bacterial strains into the more metabolically beneficial SCFA butyrate. Thus, in the current study we propose to test the effect of daily oral E. hallii treatment in 10e9/ml dose (duration 14 days) in males with type 2 diabetes treated with stable dosages of metformin (2 - 3dd 500mg or 2 dd 850mg once daily). Furthermore, a recent study showed that the amount of faecal butyrate-producing bacteria, such as eubacterium hallii, has a favorable effect on blood pressure regulation (Menni C, Eur Heart Journ 2018). In this regard, we will measure the 24-hour blood pressure of all participants before and after 2 weeks of treatment with eubactecterium hallii or placebo in order to be able to determine any improvement after treatment.

In order to test the efficacy of 2 weeks oral E. hallii treatment on (postprandial) glucose excursions subjects are asked to wear a subcutaneous continuous glucose monitor (CGM), which measures interstitial fluid glucose on certain time points, during seven days. CGMs estimates blood glucose levels with high accuracy[1].

Thus, the goal of this study is to see if there is any biological effect of E. hallii on the small intestinal microbiota, and (short and long term) glucose metabolism, so that we can see if oral E. hallii is a viable option to improve treatment efficacy. This hypothesis is in line with a recent study that suggested that (postprandial) glucose metabolism as determined by a disposable continuous glucose meter (CGM) can be directly linked to gut microbiota composition[1].

2. OBJECTIVES

In this randomized, double-blind, placebo-controlled single center study we propose to study the effect of 14 days oral E. hallii treatment on (postprandial) glucose excursions in subjects with stable metformin monotherapy treated type 2 diabetes.

Primary objective:

The primary endpoints are (postprandial) glucose excursions determined by a wearable continuous glucose monitor (CGM) during the 14 days of intervention as well as upon 2h after standardized mixed meal test in relation to SCFA levels in faeces.

Dietary intake will be monitored during the course of the study by online dietary lists.

Secondary objective:

Secondary endpoints are changes in plasma metabolites and glucose/lipids upon standardized meal in relation oral E. hallii treatment on fecal microbiota composition[12] plasma/fecal SCFA and plasma metabolites collected at baseline, week 2 and week 4. Also Daily dietary intake will be monitored during the course of the study by https://mijn.voedingscentrum.nl/nl/eetmeter

3. STUDY DESIGN

This is a randomized double blind placebo-controlled trial in which we aim to test the effect of 14 days oral E. hallii treatment. During the run in phase subjects will continue their stable dosage of metformin (2 - 3dd 500mg or 2 dd 850mg once daily) and their glucose levels will be determined by continuous glucose measurements. Afterwards, subjects will be randomised to either oral E hallii treatment or placebo during 2 weeks (see figure 5):

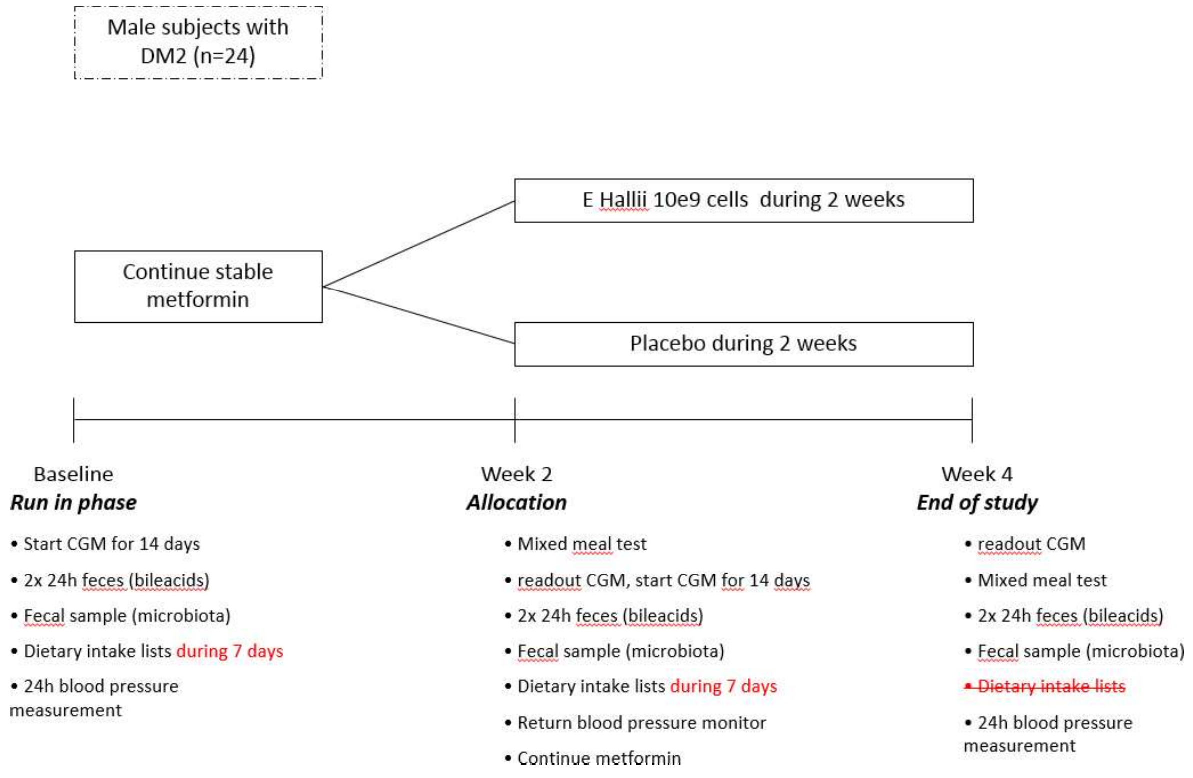

Figure 5. Study overview of the EMDM2 trial

Male Caucasian patients with type 2 diabetes treated with stable dosages of metformin (2 – 3 dd 500mg or 2 dd 850mg) will be recruited via advertisements in local magazines and at the outpatient clinic. The EMDM2 trial will be executed as follows:

<u>Screening</u>

A screening visit will be performed in the AMC, inclusion and exclusion criteria will be verified and, after oral and written explanation of the study, informed consent will be obtained. Medical history will be recorded and physical examination will take place including body weight, height, waist and hip circumference and blood pressure. The use of proton pomp inhibitors and antibiotics in the past 3 months are exclusion criteria. When a potential subject is eligible, appointments will be made for all experiments to be completed during the study.

Run in phase (baseline visit), first and second study day

The preparation for the first study day starts at home with the recording of dietary habits for the duration of the study with an online dietary booklet (https://mijn.voedingscentrum.nl/nl/eetmeter). If necessary (due to for instance long travel distance), the screeningsvisit and the baselinevisit can be combined.

After an overnight fast, subjects will visit our clinical research unit (08.30h) and bring a fresh morning fecal sample. A standardized mixed meal test (Nutridrink) will be performed at randomization and after the intervention period, with blood sampling from a placed venflon in the cubital vein during 2 hours. Also, subjects are provided with a disposable continuous glucose sensor (FreeStyle Libre)[1], which will be worn for 14 days before and 14 days after the intervention to monitor (postprandial) glucose excursions both short term and long term after the intervention. Also, they receive an blood pressure monitor to ambulatory measure their blood pressure during 24 hours before and after treatment. Moreover, subjects will be asked to collect a fresh fecal sample the next morning (t=24h) at both time points (see figure 5 for the study overview).

4. STUDY POPULATION

4.1 Population

Male Caucasian subjects with type 2 diabetes treated with stable metformin monotherapy will be recruited by local newspaper advertisements.

4.2 Inclusion criteria

In order to be eligible to participate in this study, patients must meet all of the following criteria:

- Caucasian males
- 21 to 69 years-old
- diagnosed with type 2 diabetes using oral metformin on a stable dose (i.e. no changes in the last three months), 2 - 3dd 500mg or 2 dd 850mg once daily
- no other medication use

4.3 Exclusion criteria

The following conditions are exclusion criteria for participation in this study:

- Smoking
- Alcohol abuse (>12 to 15 g of alcohol per day)
- History of cardiovascular event (myocardial infarction or pacemaker implantation)
- Cholecystectomy
- Use of any medication other than metformin, including insulin, proton pump inhibitors (PPI as this influences intestinal microbiota composition)[6], oral anticoagulants and/or oral antibiotics in the past three months
- (Expected) prolonged compromised immunity (e.g. due to recent cytotoxic chemotherapy or HIV-infection with a CD4 count < 240)
- Excessive weight loss of >10% in the last months or have overt untreated GI disease/ abnormal bowel habits.
- Levels of plasma aspartate aminotransferase and alanine aminotransferase 2.5 times or more the upper limit of the normal range

4.4 Sample size calculation

We hypothesize that a peak–difference in postprandial glucose excursions using CGM is seen between type 2 diabetes patients using placebo + metformin (13.0 mmol/l) vs E hallii + metformin (10.0 mmol/l) with a SD of 2.5 mmol/l; using unpaired t-test with 0.05 two-sided significance levels and 80% power, we will need 12 subjects per group to

detect a significant difference. Of note, all power calculations were performed with an online power calculation (www.biomath.info/power/). Based on the data retrieved from this intervention study, we will apply the prediction algorithm by Zeevi et al for validation[1]. We will include 24 males with type 2 diabetes treated with metformin. All will receive 2 weeks treatment with either E. hallii or 10% glycerol placebo.

5. TREATMENT OF SUBJECTS

5.1 Investigational product/treatment

E. hallii (produced by NIZO Bv in Wageningen according to HACCP standard, ISO9001 certified) is a Gram-positive, catalase negative bacterium belonging to the clostridial cluster XIVa of the phylum Firmicutes and is a normal inhabitant of the human intestinal tract [9]. After growth cells were harvested, washed and resuspended in PBS, supplemented with 10% glycerol and frozen at -80C. The final substance therefore comprises E. hallii cells in PBS/glycerol; also, tubes with 10% glycerol alone were produced by NIZO. This batch was also used in our previous study (METC 2014_285) and constantly kept at -80C at dept of clinical pharmacy (dr E.M. Kemper). Regular half yearly checks are done for viability/stability and purity of the vials (last check in Q4 2017, next check May 2018) see attached and in appendix 1 product dossier) and stable viability (MPN) was shown for E halli 10e9/ml dose (see figure 4).

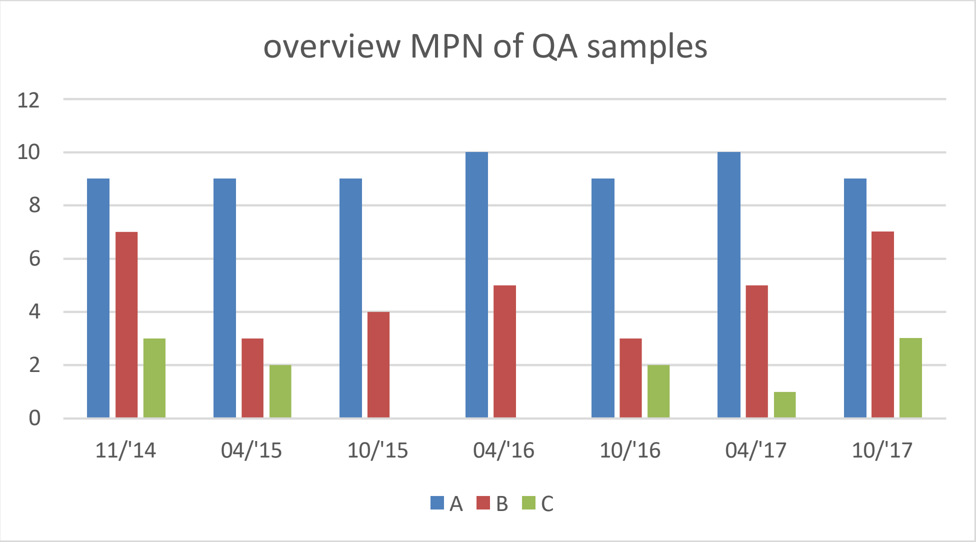

Figure 4. Viability (MPN on Ya-xis) during last years (x axis from 2014 to 2017) for E hallii 10e9/ml vial (blue bar) stored at AMC dept of clinical pharmacy.

5.2 Use of co-intervention

As a placebo 10ml of 10% glycerol will be used. Also metformin tablets will be used, but these are part of standard of care medication of all participating DM2 subjects.

6. METHODS

6.1 Study parameters/endpoints

6.1.1 Main study parameter/endpoint

(Postprandial) glucose excursions 2 hours upon standardized mixed meal test as well as two weeks after intervention (using CGM) in relation to SCFA levels in feces

6.1.2 Secondary study parameters/endpoints

Effect of oral E. hallii treatment on microbiota composition (12) and plasma metabolites determined by 16S sequencing of fecal samples collected at baseline, during week 2 and week 4.

6.2 Randomisation, blinding and treatment allocation

Subjects and treating physicians are blinded for treatment group. Randomisation of subjects will be performed by a randomisation list produced by the clinical pharmacy of the AMC. In order to prevent the distribution of participants with different doses of metformin distributed unevenly between the placebo and the intervention group, the pharmacy will apply stratification. Two groups will be made, namely one with low dose metformin (1000mg daily) and one with high dose metformin (1500mg or 1600mg daily), and these will be randomized in blocks of two. Vials with E.hallii suspension and placebo (10% glycerol) vials look similar. To maintain the blinding of the subject and investigators, the labels will be removed from the vials at the AMC pharmacy. Vials will be labelled with subject specific information prior to delivery to the study physician.

6.3 Study procedures

6.3.1 Mixed meal test (MMT)

At randomisation and 2 weeks after treatment with either E. hallii or placebo, a 2-hour standardized mixed meal test (nutridrink) will be performed after an overnight fast. For mixed meal tolerance test a venflon will be placed in the cubital vein. The mixed meal test will be a liquid solution with a standardized amount of nutrients (nutridrink 250 cc), which has to be consumed in 5 minutes and blood samples will be taken during the test[13]. Postprandial plasma samples will be taken at baseline, 10, 20, 30, 45, 60, 90 and 120minutes via an intravenous cannula (venflon) and glucose/insulin levels will be determined.

6.3.2 Continuous glucose monitoring (CGM)

Subjects will have an implanted *Freestyle Libre monitor* (http://abbottnextfrontier.com/freestyle-libre) subcutaneously that will be used for 14 days before and after the intervention to measure interstitial glucose levels[1]. Their glucose

levels will then be continuously monitored during week 1 and 2 and week 2 and 3. Subjects are not required to perform finger pricks in order to calibrate the monitor and can perform all normal activities whilst wearing a monitor.

6.3.3 24-hour ambulatory blood pressure measurements

An automatic ambulatory blood pressure monitor (Spacelabs 90207, Spacelabs In., Redmond, Washington, USA) is placed on the patient with the cuff on the non-dominant arm unless there is a 20/10 mmHg difference between arms in which case, the arm with the higher reading is used. Systolic and diastolic blood pressure and heart rate are recorded over at least 24 h. The patient's arm should be held still and at heart level during measurement. An exception is made when a measurement is being taken while a patient is engaged in certain activities, such as driving. Recordings are programmed for every 30 minutes during the day (07.00 – 22.00) and 60 minutes at night (22.00 to 07.00). These times are adjusted accordingly if the subject works at night. After 24 hours from the start of monitoring, the subject turns off and removes the monitor.
All studies are performed with the monitor display switched off, to avoid anticipation of the blood pressure readings by the patients. The ambulatory blood pressure monitoring study is only accepted when at least 70% of all measurements are successful. Mean 24-hours, daytime and night-time ambulatory SBP, DBP and heart rate will be reported.

6.3.4 24h fecal samples

Three times 2x 24h fecal samples will be collected by the subjects at baseline, week 2 and week 4 in order to study the gut microbiota. Patients will be collecting their stools in the provided materials (wearing gloves) and store it in the fridge (3-4ºC) until their visit. Samples will be transported to the AMC on icepacks.

6.4 Withdrawal of individual subjects

Subjects can leave the study at any time for any reason if they wish to do so without any consequences. The investigator can decide to withdraw a subject from the study for urgent medical reasons.

6.5 Replacement of individual subjects after withdrawal

Withdrawn subjects will be replaced during the recruitment period in order to attain the desired sample size.

6.6 Follow-up of subjects withdrawn from treatment

Withdrawn subjects will be followed for SAEs until 3 months after inclusion.

7. SAFETY REPORTING

7.1 Temporary halt for reasons of subject safety

In accordance to section 10, subsection 4, of the WMO, the sponsor will suspend the study if there is sufficient ground that continuation of the study will jeopardise subject health or safety. The sponsor will notify the accredited METC without undue delay of a temporary halt including the reason for such an action. The study will be suspended pending a further positive decision by the accredited METC. The investigator will take care that all subjects are kept informed.

7.2 AEs, SAEs and SUSARs

7.2.1 Adverse events (AEs)

E. hallii treatment from the same batch has been given in our previous study, the DIME trial (METC 2014_285). No adverse events were expected and none did. The possible complications are cited in the written patient information and are expected to be mild.

Adverse events are defined as any undesirable experience occurring to a subject during a clinical trial, whether or not considered related to the investigational drug. All adverse events reported spontaneously by the subject or observed by the investigator or his staff will be recorded.

7.2.2 Serious adverse events (SAEs)

A serious adverse event is any untoward medical occurrence or effect that

- results in death;
- is life threatening (at the time of the event);
- requires hospitalisation or prolongation of existing inpatients' hospitalisation;
- results in persistent or significant disability or incapacity;
- is a congenital anomaly or birth defect; or
- any other important medical event that did not result in any of the outcomes listed above due to medical or surgical intervention but could have been based upon appropriate judgement by the investigator.

An elective hospital admission will not be considered as a serious adverse event.

All SAEs will be reported to the accredited METC that approved the protocol, according to the requirements of that METC.

7.3 Follow-up of adverse events

All AEs will be followed until they have abated, or until a stable situation has been reached. Depending on the event, follow up may require additional tests or medical procedures as indicated, and/or referral to the general physician or a medical specialist. SAEs need to be reported till end of study within the Netherlands, as defined in the protocol

8. STATISTICAL ANALYSIS

The primary outcome is based on postprandial glycemic excursion at baseline, at randomization and 2 weeks after the intervention upon MMT to analyze metabolic control per subgroup rate (using AUC) followed by MANOVA analysis with Bonferroni testing to correct for multiple testing). Secondary measurements are tested by paired t-test or Chi Square. Differences in relative abundance of fecal bacterial strains (generated by 16S metagenomic sequencing done at the University of Gothenburg) will also be analyzed per subgroup using uni/multivariate analyses. Oral and fecal microbiota composition will be determined at the Wallenberg laboratory for Microbiology, Gothenburg, Sweden. Plasma metabolites will be determined by Metabolon.
All data will be analyzed using R Statistics (open source software) or SPSS for Windows, version 20.0 (SPSS Inc. Chicago, Illinois, USA).

9. ETHICAL CONSIDERATIONS

9.1 Regulation statement

The study will be conducted according to the principles of the Declaration of Helsinki (Fortalezea Brazil, October 2013) and in accordance with the Medical Research Involving Human Subjects Act (WMO).

9.2 Recruitment and consent

Male patients with type 2 diabetes treated with metformin will be recruited via advertisements in local magazines and at the outpatient clinic. Inclusion and exclusion criteria will be verified and, after oral and written explanation of the study, informed consent will be obtained.

9.3 Compensation for injury

The sponsor/investigator has a liability insurance which is in accordance with article 7, subsection 6 of the WMO.
The sponsor (also) has an insurance which is in accordance with the legal requirements in the Netherlands (Article 7 WMO and the Measure regarding Compulsory Insurance for Clinical Research in Humans of 1ste July 2015). This insurance provides cover for damage to research subjects through injury or death caused by the study.

1. € 650.000,-- (i.e. six hundred and fifty thousand Euro) for f injury for each subject who participates in the Research;
2. € 5.000.000,-- (i.e. five million Euro) for death or injury for all subjects who participate in the Research;
3. € 7.500.000,-- (i.e. seven million five hundred thousand Euro) for the total damage incurred by the organisation for all damage disclosed by scientific research for the Sponsor as 'verrichter' in the meaning of said Act in each year of insurance coverage

The insurance applies to the damage that becomes apparent during the study or within 4 years after the end of the study.

9.4 Incentives

All participants will receive a financial compensation of 300 euro and a refund for travel costs.

10. ADMINISTRATIVE ASPECTS, MONITORING AND PUBLICATION

10.1 Handling and storage of data and documents

The principle investigator will maintain a signature list of appropriately qualified persons to whom he delegated study duties. The investigator will maintain all eCRF's, and all source documents that support the data collected from each subject and all study documents, and will treat these data with confidentially according to the Dutch Personal Data Protection Act. Data will be entered directly in the eCRF, also verification of the eligibility criteria, medication use and adverse events are directly entered in the eCFR. A subject identification code list will be drawn, and per subject data will be filed under the subject's unique code. The subject will be identified by this number for the duration of the trial. All the participating investigators have access to the source data. Blood samples for analysis and storage will be labelled with this code and visit number. Codes cannot be retraced to the corresponding subject without the identification code list. All essential documentation and human materials will be retained by the institution. Study documents will be archived for 15 years.

10.2 Amendments

A 'substantial amendment' is defined as an amendment to the terms of the METC application, or to the protocol or any other supporting documentation, that is likely to affect to a significant degree:

- the safety or physical or mental integrity of the subjects of the trial;
- the scientific value of the trial;
- the conduct or management of the trial; or
- the quality or safety of any intervention used in the trial.

All substantial amendments will be notified to the METC and to the competent authority.

Non-substantial amendments will not be notified to the accredited METC and the competent authority, but will be recorded and filed by the sponsor.

11. REFERENCES

1. Zeevi D, Korem T, Zmora N, et al. Personalized Nutrition by Prediction of Glycemic Responses. *Cell*. 2015;163(5):1079-1095. doi:10.1016/j.cell.2015.11.001

2. Boyle JP, Thompson TJ, Gregg EW, Barker LE, Williamson DF. Projection of the year 2050 burden of diabetes in the US adult population: Dynamic modeling of incidence, mortality, and prediabetes prevalence. *Popul Health Metr*. 2010;8(1):29. doi:10.1186/1478-7954-8-29

3. Claesson MJ. for the Exploration of Microbial Gut Communities. 2010;(August):277-278. doi:10.1371/jour-nal.pone.0006669.he

4. Ley RE, Backhed F, Turnbaugh P, Lozupone CA, Knight RD, Gordon JI. Obesity alters gut microbial ecology. *Proc Natl Acad Sci*. 2005;102(31):11070-11075. doi:10.1073/pnas.0504978102

5. Ley RE. Obesity and the human microbiome. *Curr Opin Gastroenterol*. 2010;26(1):5-11. doi:10.1097/MOG.0b013e328333d751

6. Vrieze A, Holleman F, Zoetendal EG, De Vos WM, Hoekstra JBL, Nieuwdorp M. The environment within: How gut microbiota may influence metabolism and body composition. *Diabetologia*. 2010;53(4):606-613. doi:10.1007/s00125-010-1662-7

7. Vrieze A, Van Nood E, Holleman F, et al. Transfer of intestinal microbiota from lean donors increases insulin sensitivity in individuals with metabolic syndrome. *Gastroenterology*. 2012;143(4):913-916.e7. doi:10.1053/j.gastro.2012.06.031

8. Louis P, Young P, Holtrop G, Flint HJ. Diversity of human colonic butyrate-producing bacteria revealed by analysis of the butyryl-CoA:acetate CoA-transferase gene. *Environ Microbiol*. 2010;12(2):304-314. doi:10.1111/j.1462-2920.2009.02066.x

9. Duncan SH, Louis P, Flint HJ. Lactate-Utilizing Bacteria , Isolated from Human Feces , That Produce Butyrate as a Major Fermentation Product Lactate-Utilizing Bacteria , Isolated from Human Feces , That Produce Butyrate as a Major Fermentation Product. *App*. 2004;70(10):5810-5817. doi:10.1128/AEM.70.10.5810

10. Udayappan S, Manneras-Holm L, Chaplin-Scott A, et al. Oral treatment with Eubacterium hallii improves insulin sensitivity in db/db mice. *npj Biofilms Microbiomes*. 2016;2(May). doi:10.1038/npjbiofilms.2016.9

11. Parséus A, Sommer N, Sommer F, et al. Microbiota-induced obesity requires farnesoid X receptor. *Gut*. 2017;66(3):429-437. doi:10.1136/gutjnl-2015-310283

12. Wu H, Esteve E, Tremaroli V, et al. Metformin alters the gut microbiome of individuals with treatment-naive type 2 diabetes, contributing to the therapeutic effects of the drug. *Nat Med*. 2017;23(7):850-858. doi:10.1038/nm.4345

13. Dalla Man C, Campioni M, Polonsky KS, et al. Two-hour seven-sample oral glucose tolerance test and meal protocol: minimal model assessment of beta-cell responsivity and insulin sensitivity in nondiabetic individuals. *Diabetes*. 2005;54(11):3265-3273. doi:10.1161/ATVBAHA.113.301765

Suppl. Data S2: Consort checklist. Related to figure 1.

CONSORT 2010 checklist of information to include when reporting a randomised trial*

Section/Topic	Item No	Checklist item	Reported on page No
**Title and abstract**			
	1a	Identification as a randomised trial in the title	1
	1b	Structured summary of trial design, methods, results, and conclusions (for specific guidance see CONSORT for abstracts)	3
**Introduction**			
Background and objectives	2a	Scientific background and explanation of rationale	4
	2b	Specific objectives or hypotheses	5
**Methods**			
Trial design	3a	Description of trial design (such as parallel, factorial) including allocation ratio	6
	3b	Important changes to methods after trial commencement (such as eligibility criteria), with reasons	6
Participants	4a	Eligibility criteria for participants	6
	4b	Settings and locations where the data were collected	6
Interventions	5	The interventions for each group with sufficient details to allow replication, including how and when they were actually administered	6-7
Outcomes	6a	Completely defined pre-specified primary and secondary outcome measures, including how and when they were assessed	3
	6b	Any changes to trial outcomes after the trial commenced, with reasons	NA
Sample size	7a	How sample size was determined	7
	7b	When applicable, explanation of any interim analyses and stopping guidelines	NA
Randomisation:			
Sequence generation	8a	Method used to generate the random allocation sequence	7
	8b	Type of randomisation; details of any restriction (such as blocking and block size)	7
Allocation concealment mechanism	9	Mechanism used to implement the random allocation sequence (such as sequentially numbered containers), describing any steps taken to conceal the sequence until interventions were assigned	7
Implementation	10	Who generated the random allocation sequence, who enrolled participants, and who assigned participants to interventions	7
Blinding	11a	If done, who was blinded after assignment to interventions (for example, participants, care providers, those	7

		assessing outcomes) and how	
	11b	If relevant, description of the similarity of interventions	NA
Statistical methods	12a	Statistical methods used to compare groups for primary and secondary outcomes	7
	12b	Methods for additional analyses, such as subgroup analyses and adjusted analyses	7
**Results**			
Participant flow (a diagram is strongly recommended)	13a	For each group, the numbers of participants who were randomly assigned, received intended treatment, and were analysed for the primary outcome	12
	13b	For each group, losses and exclusions after randomisation, together with reasons	12
Recruitment	14a	Dates defining the periods of recruitment and follow-up	12
	14b	Why the trial ended or was stopped	12
Baseline data	15	A table showing baseline demographic and clinical characteristics for each group	12
Numbers analysed	16	For each group, number of participants (denominator) included in each analysis and whether the analysis was by original assigned groups	12
Outcomes and estimation	17a	For each primary and secondary outcome, results for each group, and the estimated effect size and its precision (such as 95% confidence interval)	12
	17b	For binary outcomes, presentation of both absolute and relative effect sizes is recommended	NA
Ancillary analyses	18	Results of any other analyses performed, including subgroup analyses and adjusted analyses, distinguishing pre-specified from exploratory	NA
Harms	19	All important harms or unintended effects in each group (for specific guidance see CONSORT for harms)	12
**Discussion**			
Limitations	20	Trial limitations, addressing sources of potential bias, imprecision, and, if relevant, multiplicity of analyses	18
Generalisability	21	Generalisability (external validity, applicability) of the trial findings	18
Interpretation	22	Interpretation consistent with results, balancing benefits and harms, and considering other relevant evidence	15-16
**Other information**			
Registration	23	Registration number and name of trial registry	6
Protocol	24	Where the full trial protocol can be accessed, if available	NA
Funding	25	Sources of funding and other support (such as supply of drugs), role of funders	21

*We strongly recommend reading this statement in conjunction with the CONSORT 2010 Explanation and Elaboration for important clarifications on all the items. If relevant, we also recommend reading CONSORT extensions for cluster randomised trials, non-inferiority and equivalence trials, non-pharmacological treatments, herbal interventions, and pragmatic trials. Additional extensions are forthcoming: for those and for up to date references relevant to this checklist, see www.consort-statement.org.
